# P75 neurotrophin receptor controls subventricular zone neural stem cell migration after stroke

**DOI:** 10.1007/s00441-021-03539-z

**Published:** 2021-10-26

**Authors:** Sachin S. Deshpande, Subash C. Malik, Pasquale Conforti, Jia-di Lin, Yu-Hsuan Chu, Suvra Nath, Franziska Greulich, Meike-Ast Dumbach, N. Henriette Uhlenhaut, Christian Schachtrup

**Affiliations:** 1 Institute of Anatomy and Cell Biology, Faculty of Medicine, University of Freiburg, Freiburg, Germany; 2 Faculty of Biology, University of Freiburg, Freiburg, Germany; 3 Molecular Endocrinology, IDC, Helmholtz Diabetes Center (HMGU) and German Center for Diabetes Research (DZD), Munich, Germany; 4 Metabolic Programming, TUM School of Life Sciences, Weihenstephan & ZIEL-Institute for Food & Health, Freising, Germany; 5 Center for Basics in NeuroModulation (NeuroModulBasics), Faculty of Medicine, University of Freiburg, Freiburg, Germany

**Keywords:** Neurotrophin receptor, Ischemic stroke, Vascular damage, Bone morphogenetic protein, Stem cell migration, Cytoskeleton

## Abstract

Stroke is the leading cause of adult disability. Endogenous neural stem/progenitor cells (NSPCs) originating from the subventricular zone (SVZ) contribute to the brain repair process. However, molecular mechanisms underlying CNS disease-induced SVZ NSPC-redirected migration to the lesion area are poorly understood. Here, we show that genetic depletion of the p75 neurotrophin receptor (*p75*^*NTR−/−*^) in mice reduced SVZ NSPC migration towards the lesion area after cortical injury and that *p75*^*NTR−/−*^ NSPCs failed to migrate upon BDNF stimulation in vitro. Cortical injury rapidly increased p75^NTR^ abundance in SVZ NSPCs via bone morphogenetic protein (BMP) receptor signaling. SVZ-derived *p75*^*NTR−/−*^ NSPCs revealed an altered cytoskeletal network- and small GTPase family-related gene and protein expression. In accordance, BMP-treated non-migrating *p75*^*NTR−/−*^ NSPCs revealed an altered morphology and α-tubulin expression compared to BMP-treated migrating wild-type NSPCs. We propose that BMP-induced p75^NTR^ abundance in NSPCs is a regulator of SVZ NSPC migration to the lesion area via regulation of the cytoskeleton following cortical injury.

**Supplementary Information:**

The online version contains supplementary material available at 10.1007/s00441-021-03539-z.

## Introduction

There is limited brain repair and recovery after stroke. Neural stem/progenitor cells (NSPCs) originating from the subventricular zone (SVZ) are an endogenous source for cell replacement and brain repair (Benner et al. 2013; Faiz et al. 2015; Pous et al. 2020). Pathological states, such as stroke, induce dynamic changes in the NSPC niche environment and trigger a regenerative response from the SVZ niche, leading to increased proliferation of NSPCs, their redirected migration towards the lesion area, and potential cell engraftment (Arvidsson et al. 2002; Kojima et al. 2010; Thored et al. 2006). However, the underlying mechanisms of SVZ NSPC activation, migration, differentiation, and cell engraftment are still poorly understood.

Under homeostatic conditions, a fine-tuned cellular and molecular niche environment coordinates continuous SVZ neurogenesis. In the adult rodent brain SVZ, astrocyte-like type B stem cells generate type C transit amplifying cells and subsequently doublecortin-positive (DCX +) neuroblasts. We collectively refer to B and C cells as NSPCs. Neuroblasts leave the SVZ and migrate long distances through the rostral migratory stream to the olfactory bulb (OBs) to become interneurons (Doetsch et al. 1999; Lois and Alvarez-Buylla 1994; Obernier and Alvarez-Buylla 2019). Upon cortical brain injury, the altered cell- or blood-derived signals from the injury site are sought to attract the SVZ-derived NSPCs to migrate towards the lesion area contributing to brain repair (Kojima et al. 2010; Ohab et al. 2006; Thored et al. 2007). Upon stroke, SVZ-derived NSPCs of the adult SVZ primarily differentiate into astrocytes and contribute to brain repair at the lesion site (Benner et al. 2013; Faiz et al. 2015; Pous et al. 2020). Understanding the molecular mechanisms that mediate the initiation of migration of newly born SVZ-derived NSPCs towards the cortical lesion area after stroke is instrumental for harnessing these cells for brain repair.

The p75 neurotrophin receptor (p75^NTR^), a member of the tumor necrosis factor receptor (TNFR) superfamily, participates in multiple intracellular signaling pathways to regulate a wide range of biological functions that can either promote or inhibit the overall process of tissue repair (Barker 2004; Dechant and Barde 2002; Hempstead 2002; Malik et al. 2021). P75^NTR^ is expressed in the nervous system in adult hippocampal (Bernabeu and Longo 2010; Catts et al. 2008; Colditz et al. 2010) and SVZ NSPCs (Young et al. 2007). The p75^NTR^ expression has been shown to define a population of neurotrophin-responsive neurogenic NSPCs and p75^NTR^ is suggested to regulate neurotrophin-fostered neuroblast migration in the rostral migratory stream towards the OBs to ensure proper neurogenesis in the healthy brain (Snapyan et al. 2009; Young et al. 2007). However, the role for p75^NTR^ in the CNS disease-induced SVZ-derived NSPC migration towards the cortical lesion site is unknown. Here, we show that p75^NTR^ is necessary for SVZ-derived newborn NSPC migration towards the cortical lesion area after stroke. P75^NTR^ is rapidly upregulated in SVZ NSPCs via bone morphogenetic protein (BMP) receptor signaling. *P75*^*NTR−/−*^ NSPCs showed an altered cytoskeleton organization, revealed by differential mRNA expression related to the activation status of the small GTPase family and cytoskeletal network changes, potentially leading to an impaired BDNF-mediated migration. Mechanistically, the altered niche environment after stroke with increased BMP-2 expression rapidly induces p75^NTR^ abundance in NSPCs, which might potentially change the cytoskeletal organization in NSPCs and guiding their migration towards the injury site. Thus, we propose that p75^NTR^ serves as a molecular link between the altered SVZ stem cell niche environment and endogenous SVZ-derived NSPC contribution to brain repair by controlling NSPC migration.

## Materials and methods

### Animals

For analysis of SVZ NSPCs, *Nestin-CreER*^*T2*^ mice (Lagace et al. 2007) were crossed with *YFP*^*fl*^ mice (Srinivas et al. 2001), resulting in *Nestin-CreER*^*T2*^*;YFP*^*fl*^ mice. For analyzing the role of the p75 neurotrophin receptor (p75^NTR^) in SVZ NSPCs, *Nestin-CreER*^*T2*^*;YFP*^*fl*^ mice were crossed with *p75*^*NTR−/−*^ mice (Lee et al. 1992). C57BL/6 J‐inbred mice deficient for *p75*^*NTR*^ were used for NSPC isolation and culture. All mice were in C57Bl/6 background crossed for more than ten generations and littermates were used in all experiments. Adult mice (8–12 weeks of age) of both sexes were used for experiments. Mice were housed under a 12-h light/dark cycle. Up to five animals per cage were housed. They were fed standard chow and had access to food and water ad libitum. All animal experiments were approved by the Federal Ministry for Nature, Environment and Consumers Protection of the state of Baden-Württemberg and were performed in accordance to the respective national, federal, and institutional regulations.

### Tamoxifen treatment

For the induction of Cre recombinase activity in the adult SVZ NSPCs, tamoxifen (Sigma-Aldrich) dissolved in corn oil (20 mg/mL) was injected intraperitoneally at 180 mg/kg/day for 5 consecutive days in the *Nestin-CreER*^*T2*^*;YFP*^*fl*^ and the *p75*^*NTR−/−*^*:Nestin-CreER*^*T2*^*;YFP*^*fl*^, as previously described (Lagace et al. 2007).

### Cortical brain injury and EdU labeling regime

Photothrombotic ischemia (PT) was performed to induce stroke in the cortex of adult mice (Labat-gest and Tomasi 2013; Pous et al. 2020). Briefly, 15 min post injection of the photosensitive dye Rose Bengal (Sigma-Aldrich; 10 μl/g body weight, intraperitoneally), a cold light illuminator of 150 W intensity was applied stereotaxically (bregma 0; mediolateral [ML], − 2.4 mm according to Paxinos and Watson). The region of interest (4 mm diameter) was illuminated for 6 min, and after the light exposure was stopped, the wound was sutured. To label the proliferating cells in the SVZ, C57BL/6 and *p75*^*NTR−/−*^ mice were intraperitoneally injected with EdU (50 mg/kg body weight, Invitrogen). Mice received one injection of EdU 1 day prior to PT for the PT day 1 and day 3 experiments.

### Stereotactic injection of BDNF

*Nestin-CreER*^*T2*^*;YFP*^*fl*^ mice were used for stereotactic injection of BDNF; controls received 0.9% NaCl. The mice were anesthetized with isofluran and placed in the stereotaxic apparatus. BDNF (0.1 µg in 1 µl of 0.9% NaCl per mouse; PeproTech, #450–02) or 0.9% NaCl (as control) was slowly injected (0.2 μl/min) with a 10-μl Hamilton syringe attached to a 33-gauge needle into mouse cortex (anteroposterior [AP], 0.0 mm; mediolateral [ML], − 1.0 mm; dorsoventral [DV], − 2.00 mm from bregma according to Paxinos and Watson).

### Immunohistochemistry

Mice were transcardially perfused with ice-cold saline, followed by 4% paraformaldehyde in phosphate buffer, and 30 µm sections were cut after cryoprotection. Analysis of the brain tissue was performed as described (Schachtrup et al. 2010). For EdU detection, we used the Click-iT™ Plus EdU Imaging kit (Fisher Scientific) according to manufacturer’s protocol and for the detection of apoptotic cells, we used the ApopTag kit according to the manufacturer’s instructions (Merck). Primary antibodies used were rabbit anti-p75^NTR^ (1:500; Millipore, #1554), chicken anti-DCX (1:500; Abcam, #ab153668), goat anti-GFP (1:1000; Abcam, #ab5450), rabbit anti-GFP (1:1000; Life Technology), human anti-fibrinogen (1:2000; US BioLogical, #F4203-02F), guinea pig anti-S100ß (1:2000; SYSY Synaptic Systems, #287,004), rabbit anti-BMP-2 (1:200; Abcam, #ab6285), rabbit anti-BMP-4 (1:200; Abcam), goat anti-nestin (1:500; Santa Cruz Biotechnology), guinea pig anti-DCX (1:1000; Millipore), rabbit anti-GFAP (1:2000; Abcam), rat anti-GFAP (1:1000; Life Technologies), rabbit anti-BDNF (1:1000; Abcam, #ab108319), and sheep anti-Thbs4 (1:1000; R&D Systems, #AF7860). Secondary antibodies used included donkey antibodies to rabbit, rat, guinea pig, mouse, sheep, and goat conjugated with Alexa Fluor 488, 594, 647, or 405 (1:200, Jackson ImmunoResearch Laboratories). Sections were cover-slipped with 4′,6-diamidino-2-phenylindole (DAPI) (Southern Biotechnology).

### Culture of adult SVZ NSPCs

SVZ-derived NSPCs were isolated and cultured (Bohrer et al. 2015; Reynolds and Weiss 1992). SVZ-derived NSPCs were isolated to generate neurospheres (Reynolds and Weiss 1992). Briefly, the dissected adult mouse SVZ tissue was dissociated with 0.25% trypsin/HBSS and cells were cultured at a density of 50,000 cells in 25 cm^2^ culture flasks in Neurobasal-A medium (GIBCO) containing B27 with vitamin A, Pen/Strep (1%), GlutaMax (1%), glutamine (0.5%), rhFGF2 (20 ng/ml) (all from Invitrogen), and rhEGF (20 ng/ml; Sigma-Aldrich) for generation of the non-adherent neurosphere culture. For differentiation assays, SVZ NSPCs were plated on laminin (0.1 mg/ml, Sigma 2020) and poly-*D*-lysine (Millipore) coated eight-well glass slides (BD Falcon) in NSPC culture medium without rhFGF2 and rhEGF. NSPCs were treated with BMP-2 (0.5 ng/ml; Peprotech), BDNF (100 ng/ml; Peprotech) or FBS (1%). SVZ NSPCs were differentiated as indicated, fixed in 4% paraformaldehyde in phosphate buffer, and processed for immunocytochemistry. For P-Smad1/5/8 analysis, nuclear immunoreactivity was measured using the Zen Blue software. For inhibitory assays, cells were pretreated 1 h before BMP-2 (1 ng/ml; Peprotech) treatment with LDN193189 dihydrochloride, blocking BMP signaling by antagonizing the intracellular kinase domain of BMP type I receptors (500 nM; Sigma) (Engers et al. 2011). For cell migration assays, neurospheres were plated on laminin (1 µg/ml; Sigma-Aldrich) coated 22 × 22-mm cover glasses (Corning) and cultured in NSPC culture medium without rhFGF2 and rhEGF. NSPCs were treated after 1 h with BMP-2 (1 ng/ml; Peprotech) or BDNF (100 ng/ml; Peprotech). NSPCs migrated out of neurospheres for 6 h and were fixed in 4% paraformaldehyde in phosphate buffer and processed for immunocytochemistry. The quantification was performed as described (Morales-Garcia et al. 2011). Briefly, the farthest distance of cell migration from a neurosphere-derived cell, which is still in contact with the neurosphere, was measured from the edge of the sphere (Fig. 3e). At least 20 plated neurospheres per condition were analyzed.

### Immunocytochemistry

Cells were rinsed with ice-cold PBS, fixed in 4% PFA for 30 min at 4 °C, washed three times with PBS, permeabilized for 10 min at 4 °C in PBS plus 0.1% Triton X-100 (by volume), blocked in PBS with 5% BSA for 30 min at 4 °C, and washed three times in PBS. The primary antibodies used were rabbit anti-P-smad1/5/8 (1:1000; Cell Signaling), goat anti-nestin (1:500; Santa Cruz Biotechnology), phalloidin (1:5000; Abcam, #ab176753), anti-α-tubulin (1:1000; Sigma-Aldrich), and secondary antibodies used included donkey antibodies to rat, rabbit, and mouse conjugated with Alexa Fluor 488, 594 or FITC (1:200, Jackson ImmunoResearch Laboratories).

### RNA isolation and quantitative PCR

RNA was isolated from primary NSPCs and quantitative real-Time PCR was performed (Schachtrup et al. 2015). The following primers were used:

*p75*^*NTR*^*:* Fwd 5′-CTAGGGGTGTCCTTTGGAGGT-3′

Rev 5′-CAGGGTTCACACACGGTCT-3′

*Gapdh:* Fwd 5′-CAAGGCCGAGATGGGA-3′

Rev 5′-GGCCTCCCCCATTTGAT-3′

### RNA sequencing (RNA-Seq)

RNA isolation from primary adult *p75*^*NTR*−/−^ and WT NSPCs was performed as described previously (Pfurr et al. 2017). Briefly, the quality of the RNA was verified using an Agilent 2100 Bioanalyzer with RNA 6000 Nano Reagents (Agilent Technologies). Library preparation and rRNA depletion was performed using the TruSeq Stranded mRNA Library Prep Kit (Illumina) starting with 500 ng RNA as input for each sample. NGS data quality was assessed with FastQC (http://www.bioinformatics.babraham.ac.uk/projects/fastqc/). Gene-level quantification was performed with Salmon version 0.10.2 (Patro et al. 2017). Settings were -libType A, -gcBias, and -biasSpeedSamp 5 using the mm10 (GRCm38.p6) reference transcriptome provided by Ensembl (Cunningham et al. 2019). Gene count normalization and differential expression analysis was performed with DESeq2 version 1.22.0 (Love et al. 2014) after import of gene-level estimates with “tximport” (Soneson et al. 2015) in R (R version 3.6.1 (Team RC, 2017)). For gene annotation, Ensembl gene Ids were mapped to MGI symbols using the Bioconductor package “biomaRt” version 2.38 (Durinck et al. 2009) and genome information was provided by Ensembl (GRCm38.p6 (Cunningham et al. 2019). Genes with a minimal mean count across samples (baseMean) of 50, fold change of 1.5 × and Benjamin–Hochberg-adjusted *p* value < 0.05 were called significantly changed. Volcano plots were generated with the “ggplot2” R package (version 3.2.1) and heatmaps using the “qplots” package (version 3.0.1.1, https://github.com/talgalili/gplots/). Functional enrichment according to gene ontology was carried out using gProfiler and the plot was made considering all significantly upregulated GOTerm pathways of differentially downregulated genes (*p* value < 0.05; fold change > 1 × , top 100 genes) under “Biological Process” using ggplot2 package in R.

### Immunoblots

For the detection of FBS and BMP-induced p75^NTR^ expression by adult NSPCs, primary cells were treated with FBS (16.6%; Gibco™ 10,270,106) or BMP (3 ng/ml; Peprotech) for the indicated time points. For blocking experiments, adult NSPCs were treated with 3 ng/ml BMP (Peprotech) for 1 day and pretreated 1 h before BMP treatment with the BMP type I receptor inhibitor LDN193189 dihydrochloride (500 nM; Sigma). For the detection of ArhGap5, WT and *p75*^*NTR−/−*^ NSPCs under proliferation conditions were harvested (NSPC medium containing rhFGF2 (20 ng/ml, Invitrogen) and rhEGF (20 ng/ml, Sigma-Aldrich). NSPC cytoplasmic and nuclear fractions were prepared using the Active Motif kit (Active Motif 54,001). For detection of P-Smad1/5/8, WT and *p75*^*NTR−/−*^ NSPCs were treated with 0.5 ng/ml BMP-2 (Peprotech) for 1 h. For the detection of BDNF expression 3 days after photothrombosis, the cortical lesion was micro-dissected under the microscope and the tissue was homogenized using the Precellys 24 Tissue Homogenizer. The following primary antibodies were used: rabbit anti-p75^NTR^ (1:2000, Cell Signaling, D4B3), P-Smad1/5/8 (1:1000, Cell Signaling), rabbit anti-Smad1 (1:1000, Cell Signaling), rabbit anti-GAPDH (1:1000, Cell Signaling), rabbit ArhGap5 (1:2000, Cell Signaling), and rabbit anti-BDNF (1:1000, Abcam, #ab108319).

### ImageJ quantification

The Western blots were quantified using ImageJ (http://rsbweb.nih.gov/ij/) with the Gel analysis tool. Densitometry was performed with values for each band normalized to corresponding GAPDH loading controls from the same membrane.

### Microscopy and image acquisition and analysis

**Fig. 1:**
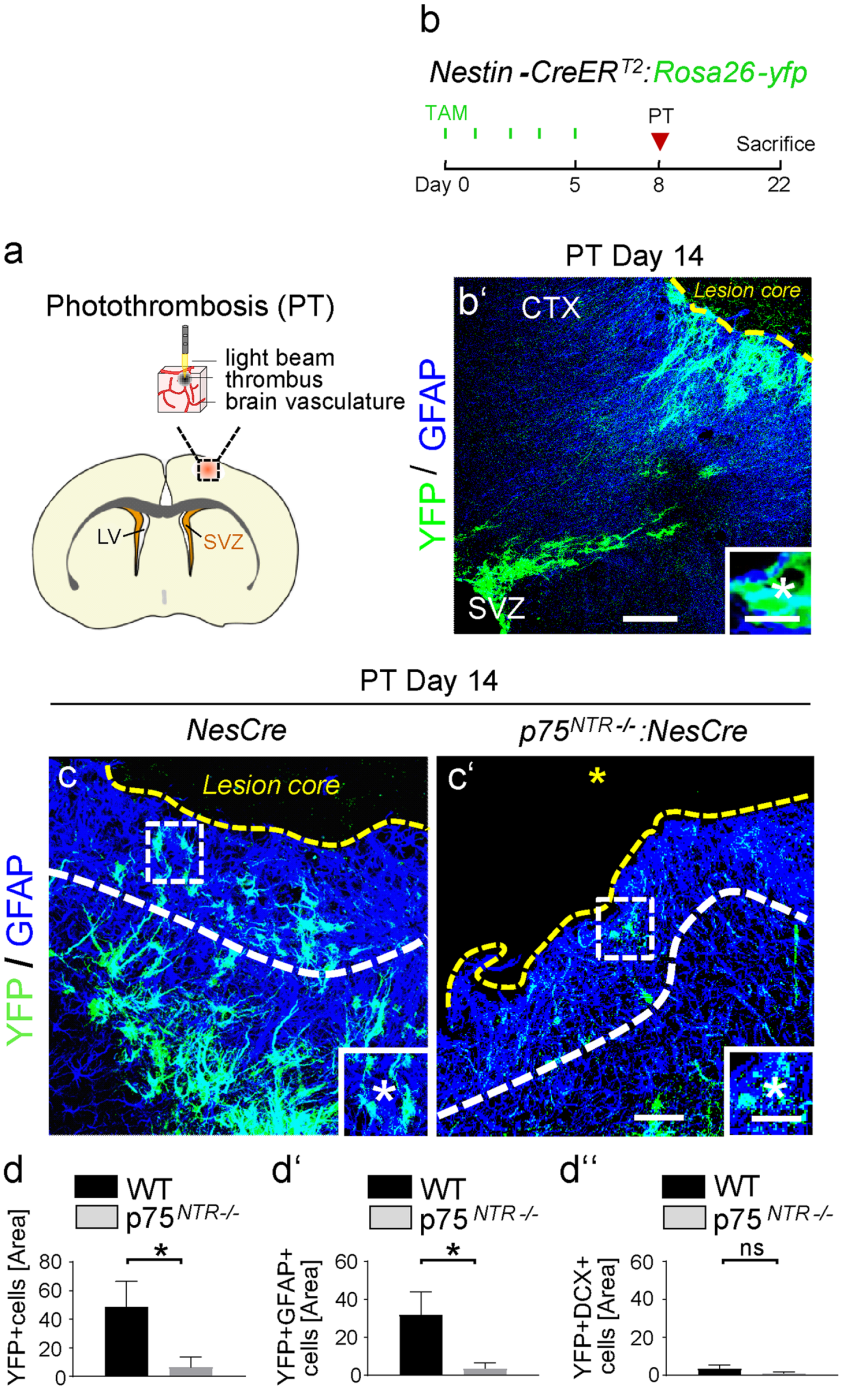
Decreased number of SVZ-derived NSPCs migrating towards the lesion area 14 days after PT in *p75*^*NTR*−/−^ mice. **a** The photothrombotic ischemia (PT) mouse model for cortical ischemic stroke. **b** The experimental setup for photothrombosis using the *Nestin-CreER*^*T2*^*;Rosa26-yfp* mice. **b ‘** Low-magnification image showing immunolabeling for YFP (green) in combination with GFAP (blue) in coronal brain sections of adult mice 14 days after PT. Enlarged image at the bottom showing SVZ-derived newborn astrocyte (asterisk) in the cortical lesion area. Distinction between lesion core and penumbra by dashed yellow line (adapted from Wanner et al. 2013 (Wanner et al. 2013) by absence of astroglia in the lesion core. **c**, **c ‘** Representative images with immunolabeling for YFP (green) and GFAP (blue) of *p75*^*NTR−/−*^ mice compared to WT mice 14 days after PT. White boxes indicating enlargement of cells in the penumbra at the bottom. Yellow dashed line delineating the lesion core. Quantification area between yellow and white dashed line in the penumbra. **d**, **d ‘‘** Quantification of YFP + , YFP + GFAP + , and YFP + DCX + SVZ-derived cells in the penumbra area per section. Scale bars = 100 μm (**b‘**), 7 μm (**b ‘** enlargement), 80 μm (**c**, **c‘**), 20 μm (**c**, **c ‘** enlargement). Values are mean ± SEM. (*p* values calculated by Student’s *t* test; ns, not significant, *p < 0.05. *n* = 4 (**d**, **d ‘‘** WT mice), *n* = 5 (**d**, **d ‘‘** *p75*^*NTR−/−*^ mice). CTX, cortex; SVZ, subventricular zone

For microscopy and image acquisition, Leica TCS SP8 Confocal and Axioplan 2 Imaging epifluorescence were used. For colocalization analysis, images from sagittal and coronal brain sections were acquired with a Leica TCS SP8 laser confocal microscope with 20 × and 40 × oil immersion objectives. Images were generated from z-stack projections (0.5–1.0 µm per stack) through a distance of 15–18 µm per brain section. The colocalization of different markers was analyzed with the LAS AF analysis software by displaying the z-stacks as maximum intensity projections and using axis clipping and the rotation of the 3D-rendered images. For immunoreactivity (IR) analysis, the Leica software was used, and total IR was calculated as percentage area density defined as the number of pixels (positively stained areas) in a multiple projection image divided by the total number of stacks in the imaged field. For colocalization and immunoreactivity analysis, images were analyzed and averaged on at least 4 randomly selected brain sections in the SVZ and in a defined area in the cortical lesion. A white dashed line (Fig. 1c) was drawn at 200 µm distance from the lesion center (yellow dashed line). Quantification of cells was performed within this area per section. For immunoreactivity analysis of α-tubulin in neurospheres, images were acquired with a Leica TCS SP8 laser confocal microscope with 63 × with 2 × zoom and total immunoreactivity per area was calculated using the LasX software on a total of 20 neurospheres. For cell culture assays, images used for quantifications were acquired with an Axioplan 2 Imaging epifluorescence microscope with 20 × and 40 × objective and analyzed with the AxioVision image analysis software (Carl Zeiss). The images were quantified with Zen Blue. To quantify nuclear P-Smad1/5/8 immunoreactivity in SVZ NSPCs, DAPI + nuclei were selected and quantified using Zen Blue. At least 80 nuclei per image were quantified and the P-Smad1/5/8 immunoreactivity values were averaged. The representative images were acquired with a Leica TCS SP8 laser confocal microscope with 10 × , 20 × , and 63 × oil immersion objectives. Images were generated from z-stack projections (0.35–1.0 µm per stack) through a distance of 1–12 µm.

### Data availability

RNA-Seq data reported in this paper are available with the GEO accession number GSE152396. Source data are provided as a source data file. All other data are available from the corresponding author upon reasonable request.

### Statistical Analysis

Data are shown as means ± SEM. Differences between groups were examined by one-way ANOVA for multiple comparisons, followed by Bonferroni’s correction for comparison of means. Differences between isolated pairs were examined by Student’s *t* test.

## Results

### Genetic depletion of p75^NTR^ reduces SVZ-derived NSPC migration to the lesion area after cortical injury

**Fig. 2:**
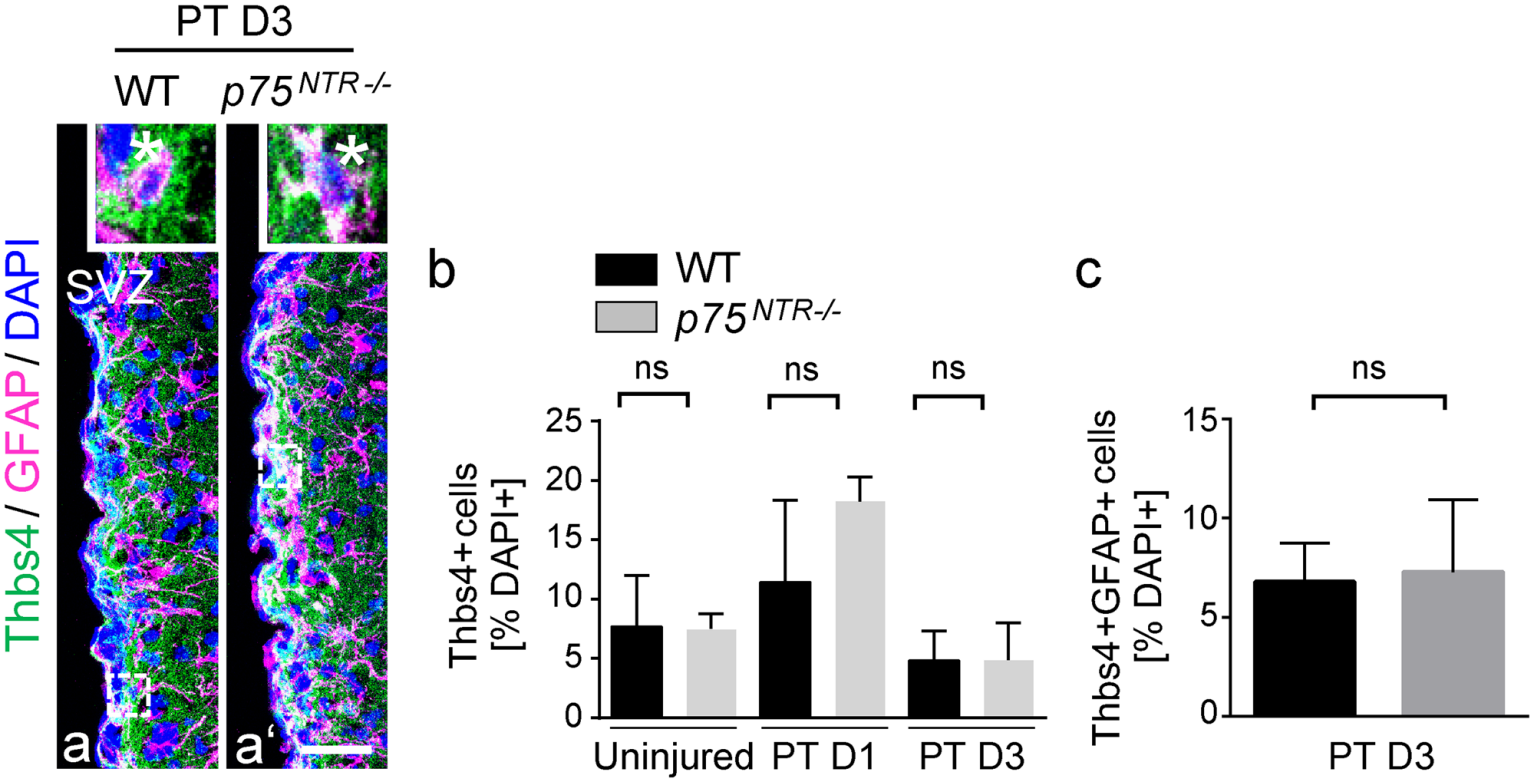
No changes in Thbs4 cells in the SVZ after PT in *p75*^*NTR*−/−^ mice. **a**, **a’** Representative images with immunolabeling for Thbs4 (green) in combination with GFAP (magenta) in the SVZ of *p75*^*NTR−/−*^ mice compared to WT mice 3 days after PT. Enlargement of region indicated by rectangles depicting a representative Thbs4 + GFAP + cell 3 days after PT (top, white star indicating a double-positive cell). **b** Quantification of the % of Thbs4 + cells in the SVZ at D1 and D3 after PT. **c** Quantification of the % of Thbs4 + GFAP + cells in SVZ at D3 after PT. Scale bars = 31 μm (**a**, **a‘**). *n* = 2–4 (**b**), *n* = 4 (**c**). SVZ, subventricular zone

After injury, NSPCs originating from the SVZ change their migration path. Instead of migrating to the OBs and becoming neurons, they migrate towards the lesion area and differentiate mainly into astrocytes (Benner et al. 2013; Pous et al. 2020). To investigate the role of p75^NTR^ in the regulation of SVZ NSPC migration towards the lesion area, we established the photothrombotic ischemia stroke model (Labat-gest and Tomasi 2013) (Fig. 1a) and used the tamoxifen-inducible *Nestin-CreER*^*T2*^*;YFP*^*fl*^ reporter transgenic mice (Lagace et al. 2007) for cell tracking of SVZ-derived NSPCs to the cortical lesion area (Fig. 1b, b ‘). Immunolabeling of SVZ-derived YFP + (green) cells in combination with either GFAP (blue), labeling SVZ-derived newborn astrocytes or DCX (blue), labeling newborn neuroblasts, and in combination with p75^NTR^ (red), revealed p75^NTR^ expression in these migrating cells (Fig. S1a, b), suggesting the p75^NTR^ might be involved in the migration of SVZ NSPCs towards the lesion area after photothrombosis (PT). Remarkably, loss of p75^NTR^ using the *p75*^*NTR−/−*^ mouse line, revealed a ~ 90% reduction of SVZ-derived YFP + cells and a ~ 85% reduction in the number of SVZ-derived newborn astrocytes (YFP + GFAP +) in the lesion area compared to control mice, while only very few SVZ-derived neuroblasts (YFP + DCX +) migrated to the lesion area (Fig. 1c, d ‘‘). PT evokes rapid changes of the SVZ NSPC fate (Pous et al. 2020). To rule out a potential role of p75^NTR^ in SVZ NSPC proliferation, apoptosis and differentiation causative for the reduced number of SVZ-derived NSPCs in the lesion area, we next analyzed the NSPC behavior within the SVZ immediately after PT. No significant differences in newborn Thbs4 + astrocyte formation, cell death, and proliferation in the SVZ stem cell niche between *p75*^*NTR−/−*^ and WT mice after PT was detected (Figs. 2 and S1c, d).

**Fig. 3:**
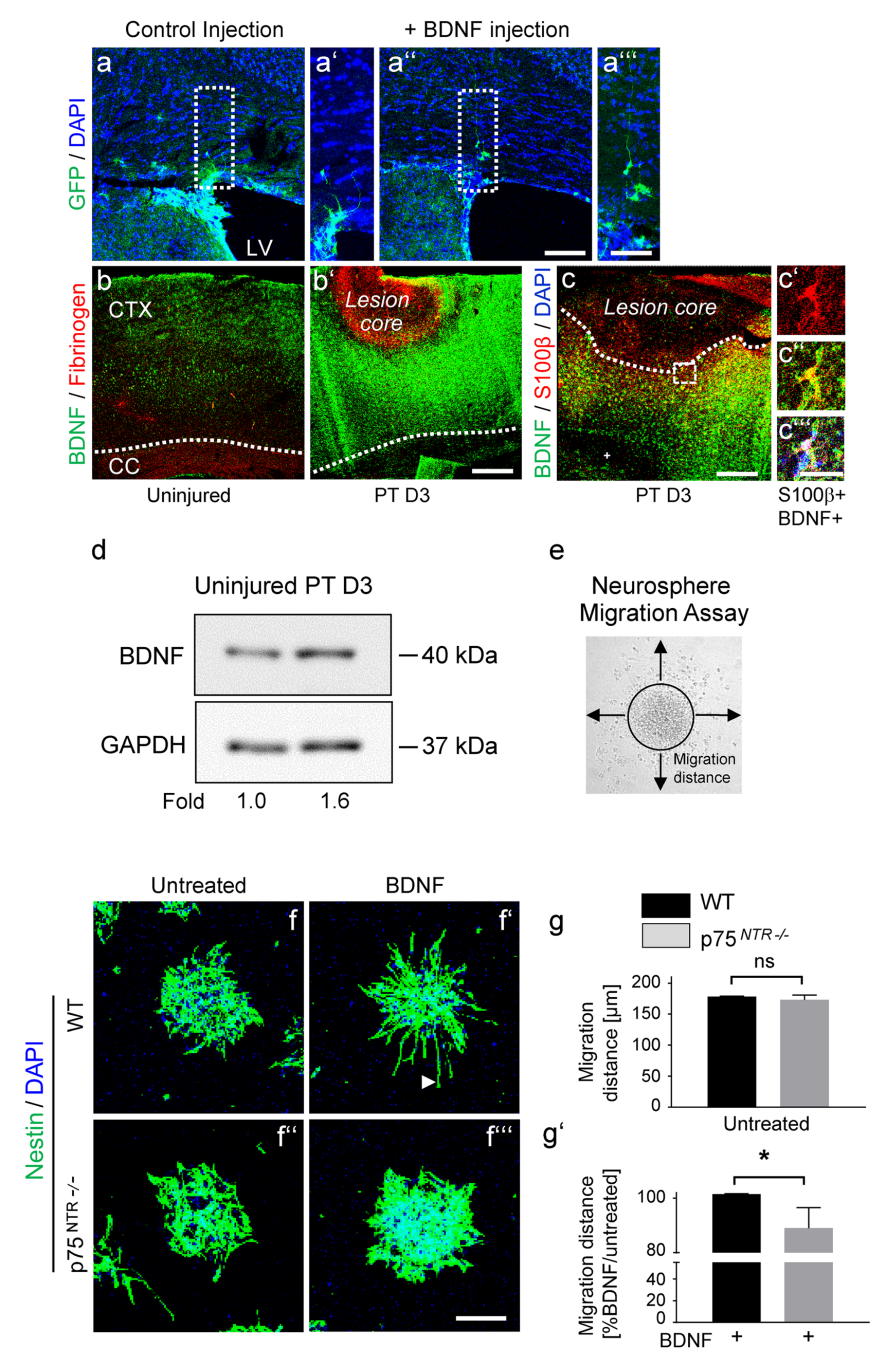
Increased BDNF expression in the cortical lesion after PT. **a**, **a ‘‘‘** Low‐magnification images showing immunolabeling for YFP (green) in coronal brain sections at day 5 after BDNF injection into the cortex compared to control mice. Enlarged image at the right showing SVZ-derived YFP + cell migrating towards the cortical BDNF injection site compared to the control injection. **b**, **b ‘** Low‐magnification images showing immunolabeling for BDNF (green) in combination with fibrinogen (red) in coronal brain sections at day 3 after PT compared to uninjured adult mice. **c**, **c ‘‘‘** Immunolabeling for BDNF (green) in combination with S100β (red) in the cortex at day 3 after PT. White box indicating S100β + BDNF + cell in the penumbra at day 3 after PT (right, enlargement) after PT. **d** Immunoblot for BDNF and GAPDH on lysate obtained from the cortical lesion area 3 days after PT compared to lysate obtained from cortex of uninjured mice. **e** The measurement of the distance of a neurosphere-derived cell. **f**, **f ‘‘‘** Immunolabeling for nestin (green) of BDNF treated *p75*^*NTR−/−*^ and WT neurospheres in vitro 6 h after plating on laminin. White arrowhead indicating representative bipolar, elongated WT NSPC that migrated out of the neurosphere after BDNF treatment. **g**, **g ‘** Quantification of the migration distance of untreated and BDNF treated *p75*^*NTR−/−*^ NSPCs compared to WT NSPCs. Nuclei are stained with DAPI (blue). Scale bars = 112 µm (**a**, **a ‘‘**), 56 µm (**a‘**, **a ‘‘‘**), 100 μm (**b**, **b‘**), 100 μm (**c**), 10 μm (**c‘**, **c ‘‘‘**), 80 µm (**f**, **f ‘‘‘**). Representative images are shown, *n* = 2 mice (**a**, **a ‘‘‘**), *n* = 4 mice (**b**, **c ‘‘‘**). Representative Western blots are shown, *n* = 2. A minimum of 20 neurospheres per condition were analyzed (**f**, **g‘**). CTX, cortex; CC, corpus callosum; LV, lateral ventricle

Brain-derived neurotrophic factor (BDNF) has been shown to promote SVZ neuroblast migration to the OBs (Snapyan et al. 2009) and NSPC migration in acute brain slices (Grade et al. 2013). Immunolabeling of YFP + cells 5 days after cortical BDNF injection revealed bipolar, elongated SVZ-derived YFP + cell migration towards the cortical injection site compared to the control injection, suggesting that BDNF could also act as an attractant for adult SVZ-derived p75^NTR^ + NSPC migration towards the cortical lesion area (Fig. 3a, a ‘‘‘). Immunolabeling for BDNF (green) in combination with the blood-derived protein fibrinogen (red), labelling the cortical injury site, revealed increased BDNF expression level in the ipsilateral cortex at day 3 after PT, the time point when SVZ-derived NSPCs redirect their migration towards a cortical lesion (Bohrer et al. 2015) (Fig. 3b, b ‘). Furthermore, immunolabeling of S100β + astrocytes (red) in combination with BDNF (green) revealed astrocytes as one potential source of increased BDNF after cortical injury (Fig. 3c, c‘‘‘). Immunoblot protein expression analysis for BDNF of cortical tissue at day 3 after PT further confirmed the increased BDNF protein expression level in the ipsilateral cortex at day 3 after PT (Fig. 3d; original blots are presented in Fig. S5), indicating that BDNF could be an attractant for SVZ-derived p75^NTR^ + NSPC migration towards the cortical lesion area. Importantly, *p75*^*NTR−/−*^ NSPCs are resistant to BDNF-induced migration in vitro (Fig. 3e–g‘), suggesting that p75^NTR^ increases the number of SVZ-derived NSPCs migrating towards the lesion area after cortical injury.

### P75^NTR^ expression is rapidly upregulated in SVZ NSPCs via BMP signaling after cortical injury

**Fig. 4:**
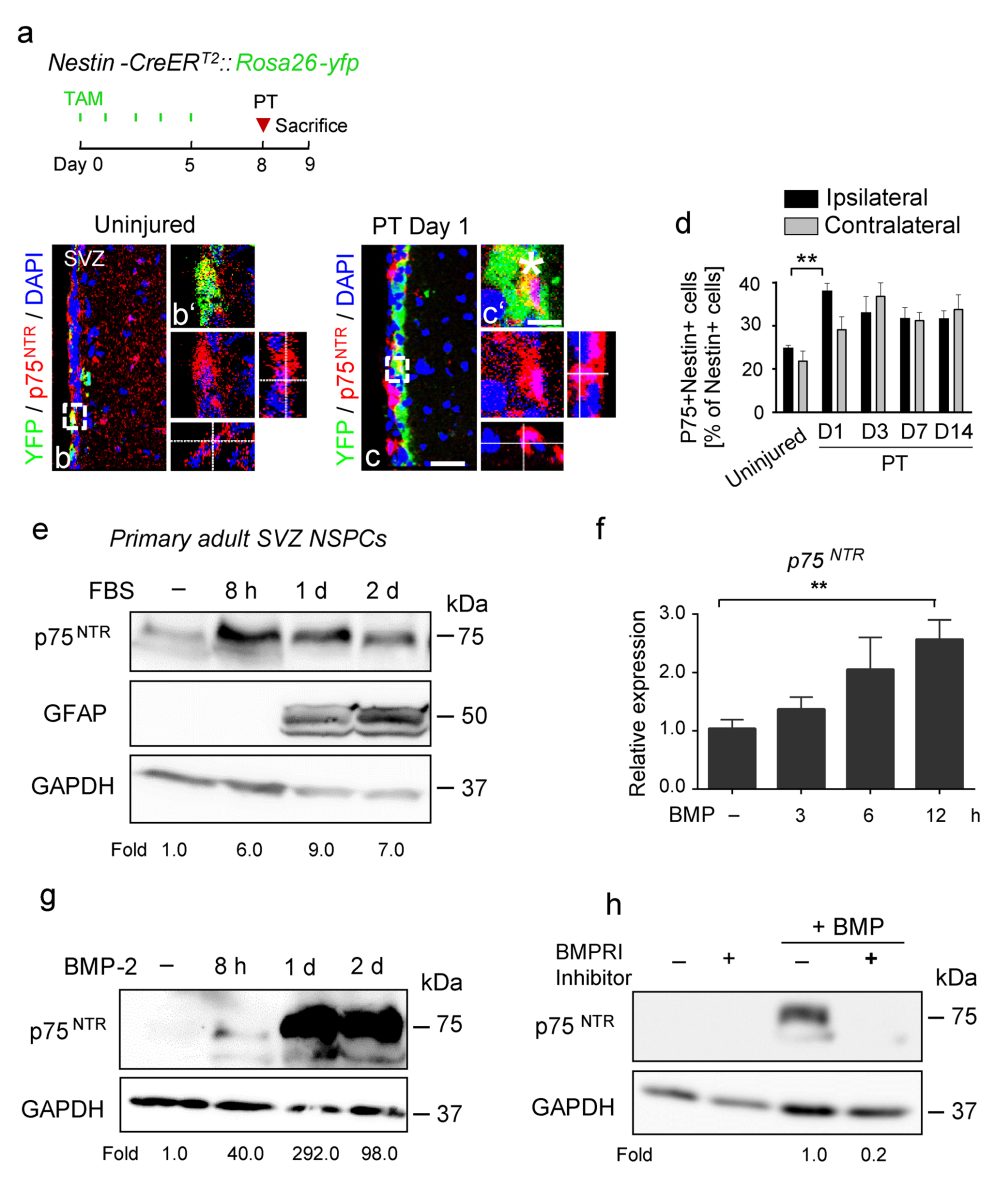
BMPR signaling-induced p75^NTR^ expression in SVZ NSPCs. **a** Scheme illustrating experimental setup for photothrombosis on *Nestin-CreER*^*T2*^*;YFP*^*fl*^ mice. **b**, **c ‘** Immunolabeling for p75^NTR^ (red) in combination with YFP (green) in the SVZ niche 1 day after PT. Enlargement of region indicated by rectangles depicting a representative p75^NTR^ + YFP + cell 1 day after PT (**c**, **c‘**, white star indicating nuclear expression pattern) compared to a representative p75^NTR^ + YFP + cell of uninjured mice (**b**, **b‘**). **d** Quantification of the number of p75^NTR^ + nestin + cells at 1, 3, 7, and 14 days after PT and uninjured control. **e** Immunoblot protein expression analysis for p75^NTR^ in FBS‐treated WT NSPCs. **f** Expression of *p75*^*NTR*^ mRNA in NSPCs after 3 h, 6 h, and 12 h of BMP‐2 treatment compared to untreated cells determined by quantitative PCR and normalized to *Gapdh*. **g** Protein expression of p75^NTR^ in NSPCs after 8 h, 1 day, and 2 days of BMP‐2 treatment determined by Western blotting. **h** Immunoblot for p75^NTR^ in NSPCs pretreated with a BMP receptor inhibitor before BMP stimulation. Nuclei are stained with DAPI (blue). Scale bars = 50 µm (**b**, **c**), 7 µm (**b‘**, **c‘**). Values are mean ± SEM (*p* values calculated by one-way ANOVA and Bonferroni’s multiple comparisons test; ns, not significant, **p < 0.01). Quantitative PCR results are of four independent experiments performed in duplicate (**f**). Representative images are shown, *n* = 3 (**b**, **c**). *n* = 4 mice per group (**d**). Representative Western blots are shown, *n* = 2 (**e**, **g**, **h**). SVZ, subventricular zone

In the healthy brain, NSPC maintenance, migration, and differentiation is instructed by the extracellular environment in their particular stem cell niche to secure continued neurogenesis. CNS injury and disease drastically changes the SVZ stem cell niche environment (Bohrer et al. 2015; Pous et al. 2020). P75^NTR^ expression is increased during a wide range of pathological conditions and its NSPC subpopulation specific expression in a pathological context might influence the receptor function. Therefore, we next addressed the temporal expression of p75^NTR^ in SVZ NSPC subpopulations after PT. The cellular composition of the adult SVZ (Zywitza et al. 2018) can be distinguished by differential marker expression (Codega et al. 2014; Ming and Song 2011), while SVZ astrocytes and adult astrocyte-like stem cells (quiescent and active B cells) are characterized by GFAP expression, active B cells and transit amplifying C cells are characterized by nestin expression and newborn young neurons (neuroblasts, A cells) are characterized by DCX expression. However, unique marker for NSPC subpopulations are still missing and the cell composition of the adult SVZ neurogenic niche changes due to an astrogenic milieu after CNS injury and disease (Pous et al. 2020). PT rapidly changed the SVZ neural stem cell niche environment, leading to a strong overall increase in GFAP + cells within the SVZ niche area and an induction of NSPC differentiation into Thbs4 + GFAP + S100β + SVZ newborn astrocytes, while the total cell number and the number of apoptotic cells did not change significantly (Pous et al. 2020). Our study showed that the number of nestin + cells (active B cells and proliferating C cells) and the number of DCX + neuroblasts (young neurons) increased significantly at day 1 and day 3 as well as at day 1 after PT, respectively (Fig. S2a, b). Importantly, the percentage of p75^NTR^ + nestin + cells among nestin + cells and the percentage of p75^NTR^ + DCX + neuroblasts among DCX + cells increased significantly at day 1 after PT compared to uninjured control animals, while the percentage of p75^NTR^ + GFAP + cells among GFAP + cells (B cells and astrocytes) decreased significantly at day 3 after PT compared to uninjured control animals. The reduced portion of p75^NTR^ + GFAP + cells among GFAP + B cells and astrocytes after PT might be masked by changes in the SVZ cell composition after injury and by a strong increase in the overall GFAP + cell number in the SVZ (newborn astrocytes, reactive niche astrocytes, re-expression of GFAP by other cells) (Pous et al. 2020). In addition, it might be possible that p75^NTR^ expression might characterize a distinct SVZ B cell or niche astrocyte subpopulation. Finally, the increased percentage of p75^NTR^ + cells among active B cells, proliferating C cells, and neuroblasts suggests a role for p75^NTR^ in the fate control of these cells at early time points after PT (Figs. 4a–d and S2c–h). Subsequently, the percentage of p75^NTR^ + cells of all investigated NSPC subpopulations returned to basal level at later time points after PT (Fig. S2c–h).

Overall, PT resulted in an ipsilateral and contralateral SVZ stem cell niche activation, with a rapid upregulation of p75^NTR^ in NSPC subpopulations (Figs. 4 and S2), suggesting prompt changes in the SVZ NSPC microenvironment that regulate p75^NTR^ after cortical brain injury. So far, the mechanisms that regulate p75^NTR^ expression in the nervous system are largely unknown.

Cortical injury results in a changed SVZ stem cell niche environment due to blood–brain barrier (BBB) opening, leakage of blood proteins into the SVZ stem cell niche, increased BMP level, and activation of BMP signaling (Bohrer et al. 2015; Pous et al. 2020). In vitro cultured primary NSPCs derived from adult SVZ mouse tissue express p75^NTR^, and FBS treatment, mimicking a changed SVZ stem cell niche environment with a strong astrogenic effect, rapidly upregulated p75^NTR^ expression (Fig. 4e). In order to investigate the impact of cortical ischemic stroke on the SVZ niche extracellular environment and its potential rapid regulation of p75^NTR^ in NSPCs, we first analyzed the abundance of BMP isoforms in the SVZ at 18 h and 36 h after PT, which are time points prior to the p75^NTR^ upregulation in adult SVZ NSPC subpopulations after cortical injury. Immunolabeling for BMP-2 (green) revealed increased BMP-2 expression in the ipsilateral SVZ at 18 h and 36 h after PT (Fig. S3a, b). Furthermore, nestin + (red) NSPCs showed an increased BMP-2 (green) expression at 18 h after PT, while extracellular BMP-2 (green) is deposited in the SVZ niche environment surrounding nestin + (red) NSPCs at 36 h after PT (Fig. S3c). BMP-4 expression was not upregulated in the SVZ at the analyzed time points after PT (data not shown). Importantly, treatment of NSPCs with BMP-2 induced a strong and rapid upregulation of *p75*^*NTR*^ mRNA expression and increased the p75^NTR^ protein abundance (Fig. 4f–g), while pretreating cells with a BMP type I receptor inhibitor blocked p75^NTR^ upregulation via BMP-2 (Fig. 4h), suggesting that increased expression level of BMP-2 in the SVZ stem cell niche after PT is responsible for the rapid increase of the p75^NTR^ abundance in SVZ NSPCs.

### P75^NTR^-regulated genes in NSPCs are associated with regulating small GTPase family activity and cytoskeletal organization

**Fig. 5:**
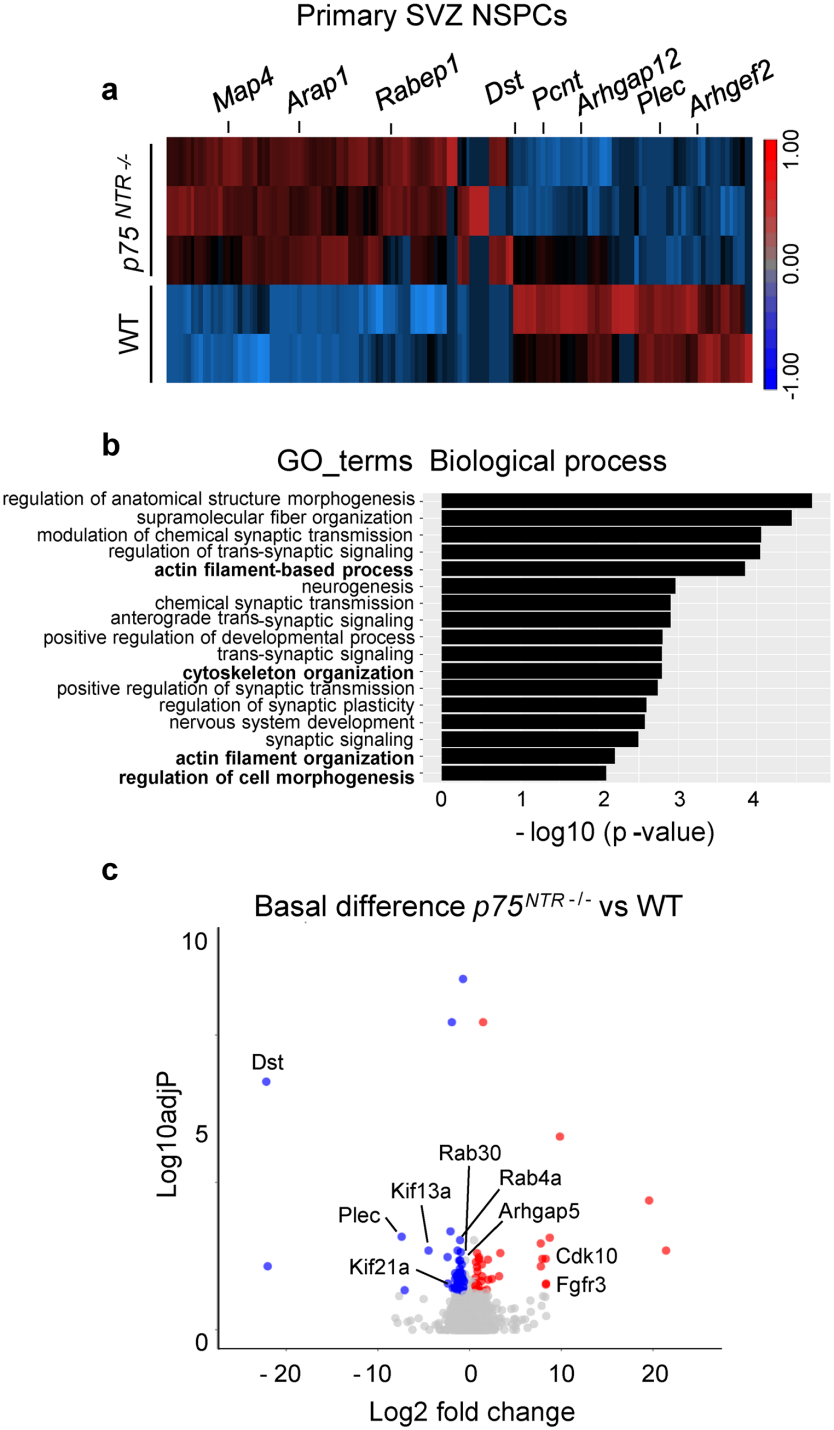
p75^NTR^-regulated genes in NSPCs are involved in cytoskeletal organization. **a** Heatmap showing genes that are differentially expressed by a factor of at least 1.5 between *p75*^*NTR−/−*^ and WT NSPCs (adjP < 0.05). Colors reflect the mean raw *z* score. Blue for lower than average and red for higher than average expression. **b** Gene ontology enrichment analysis of biological processes for genes with reduced expression in *p75*^*NTR−/−*^ compared to WT NSPCs (*p* value < 0.05; fold change > 1 ×). **c** Volcano plot of the differentially expressed genes between *p75*^*NTR−/−*^ and WT NSPCs. Red and blue points mark the genes with significantly (adjP < 0.05) increased or decreased expression, respectively in *p75*^*NTR−/−*^ compared to WT NSPC samples, while non-significant genes are in gray. The *x*-axis shows log2 fold changes in expression and the *y*-axis the negative log-transformed adjusted *p* value (adjP). Selected genes are labelled. RNA-Seq data are derived from four replicas using NSPCs from independent preparations

As a receptor for neurotrophins and an adaptor protein for multiple signaling partners, the p75^NTR^ functions at the molecular nexus of many biological processes. As an initial approach to identify which genes are regulated by p75^NTR^ during NSPC migration, we used RNA sequencing (RNA-Seq) to analyze gene expression in WT and *p75*^*NTR−/−*^ adult NSPC cultures. We clustered differentially expressed genes (adjP < 0.05, change > 1.5-fold) showing the most robust and consistent differences in expression in *p75*^*NTR*−/−^ NSPCs compared to WT control cells. Among them, we identified genes associated with cell migration (Fig. 5a and Tables S1, S2). Surprisingly, we found many genes, such as *dystonin*, *pericentrin*, *plectin*, and *haus8* that were all differentially regulated in the *p75*^*NTR−/−*^ NSPCs and are associated with cytoskeletal network organization and the microtubule system (Fig. 5a–c and Tables S1, S2, gray shadow). p75^NTR^ activates distinct signaling pathways via the p75^NTR^ ICD interaction with small GTPase family members, such as Rho and Rab (Baeza-Raja et al. 2012; Domeniconi et al. 2005; Yamashita and Tohyama 2003). Interestingly, our RNA-Seq revealed that genes associated with controlling the activation status of the small GTPase family, such as *Arhgap5*, *Arhgap12*, *Argef2*, *Rasgef1b*, and *Rabgef1* are differentially regulated in *p75*^*NTR−/−*^ NSPCs compared to WT NSPCs. To determine the biological functions of p75^NTR^ regulated genes according to enriched gene sets grouped by Gene Ontology (GO) terms, we used gProfiler. We found enrichment of the GO categories “synaptic transmission,” “development,” and “neurogenesis” suggesting a role of p75^NTR^ in the NSPC overall fate control. Moreover, we found enrichment of the GO categories “actin filament-based process,” “cytoskeletal organization,” “actin filament organization,” and “regulation of cell morphogenesis” as well as “small GTPase-mediated signal transduction” (Fig. 5b and data not shown). Several core genes responsible for cytoskeletal organization and the microtubule system, important for guiding cell migration, including *Arhgap5*, *Kif13a*, and *Plec*, are found within these enriched GO categories, suggesting that p75^NTR^ serves as a central player in controlling the cytoskeletal organization in NSPCs (Fig. 5c).

### P75^NTR^-deficient SVZ NSPCs reveal an altered morphology and cytoskeletal organization in vitro

**Fig. 6:**
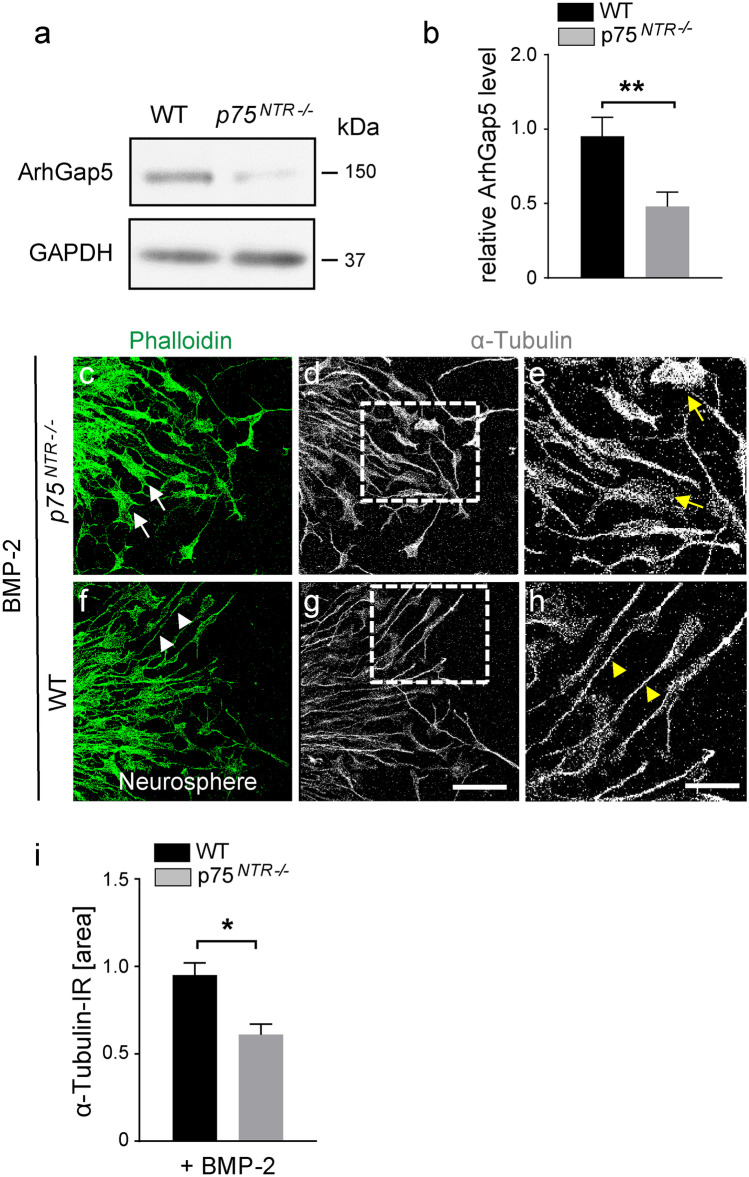
*p75*^*NTR*−/−^ SVZ NSPCs reveal and altered morphology and cytoskeletal organization. **a** Immunoblot protein expression of ArhGap5 in *p75*^*NTR−/−*^ compared to WT NSPCs. **b** Quantification graph of Arhgap5 protein expression in *p75*^*NTR−/−*^ compared to WT NSPCs. **c**–**h** Immunolabeling for phalloidin (green, left) and α-tubulin (gray, middle and enlargement right) of BMP-2 treated *p75*^*NTR−/−*^ and WT neurospheres in vitro. **i** Quantification graph of α-tubulin immunoreactivity in *p75*^*NTR−/−*^ compared to WT neurospheres. Scale bars = 35 µm (**c**, **d**, **f**, **g**), 20 µm (**e**–**h**). Values are mean ± SEM (*p* values calculated Student’s *t* test; *p < 0.05, **p < 0.01. Representative Western blot is shown, *n* = 4

Our results demonstrate that *p75*^*NTR−/−*^ NSPCs have an altered gene expression associated with cytoskeletal network organization and a disturbed migration in vivo and in vitro. Specifically, our data so far revealed that p75^NTR^ regulates small GTPase family-related mRNA expression (Fig. 5 and Tables S1, S2, gray shadow). In line with these results, *Arhgap5*, a Rho GTPase-activating protein involved in cytoskeleton changes, was reduced by 50% revealed by immunoblot protein expression of lysates from *p75*^*NTR−/−*^ NSPCs compared to WT NSPCs (Fig. 6a, b), confirming that p75^NTR^ regulates small GTPase family-related expression, in addition to the mRNA, on the protein level. The microtubule network binds to Smads, the transcriptional regulator of the BMP signaling pathway, and is involved in their nucleocytoplasmic shuttling (Dong et al. 2000). Since *p75*^*NTR−/−*^ NSPCs revealed an altered cytoskeleton organization with altered microtubule expression, we tested whether signaling molecules that interact with microtubules were affected in *p75*^*NTR−/−*^ NSPCs. BMP signals by nuclear accumulation of phosphorylated Smad (P-Smad) transcriptional regulators (Massague et al. 2005). *p75*^*NTR*−/−^ NSPCs had reduced nuclear, but not cytosolic BMP-2-dependent accumulation of P-Smad2 compared to WT (Fig. S4), suggesting a mutual dependency of BMP signaling and p75^NTR^ abundance. Finally, we tested whether the injury-induced BMPR signaling-regulated p75^NTR^ abundance affects the NSPC cytoskeleton organization, which might be required for the migration of SVZ-derived NSPCs to the cortical lesion. The cytoskeleton provides mechanical support enabling NSPC migration, which consists of microtubules, actin filaments, and intermediate filaments. In accordance, immunolabeling migrating WT NSPCs with phalloidin (green), staining the cytoskeleton via actin binding, or α-tubulin (gray) showed a typical elongated, bipolar shape (Fig. 6f–h, white and yellow arrowheads) while, in stark contrast, *p75*^*NTR−/−*^ NSPCs revealed a disorganized, flat cell morphology (Fig. 6c–e, white and yellow arrows). Moreover, *p75*^*NTR−/−*^ NSPCs treated with BMP-2 revealed a reduced α-tubulin expression compared to wt cells (Fig. 6i), suggesting that p75^NTR^ abundance equips SVZ NSPCs for cell migration towards the lesion area via regulating the cytoskeleton organization.

## Discussion

**Fig. 7:**
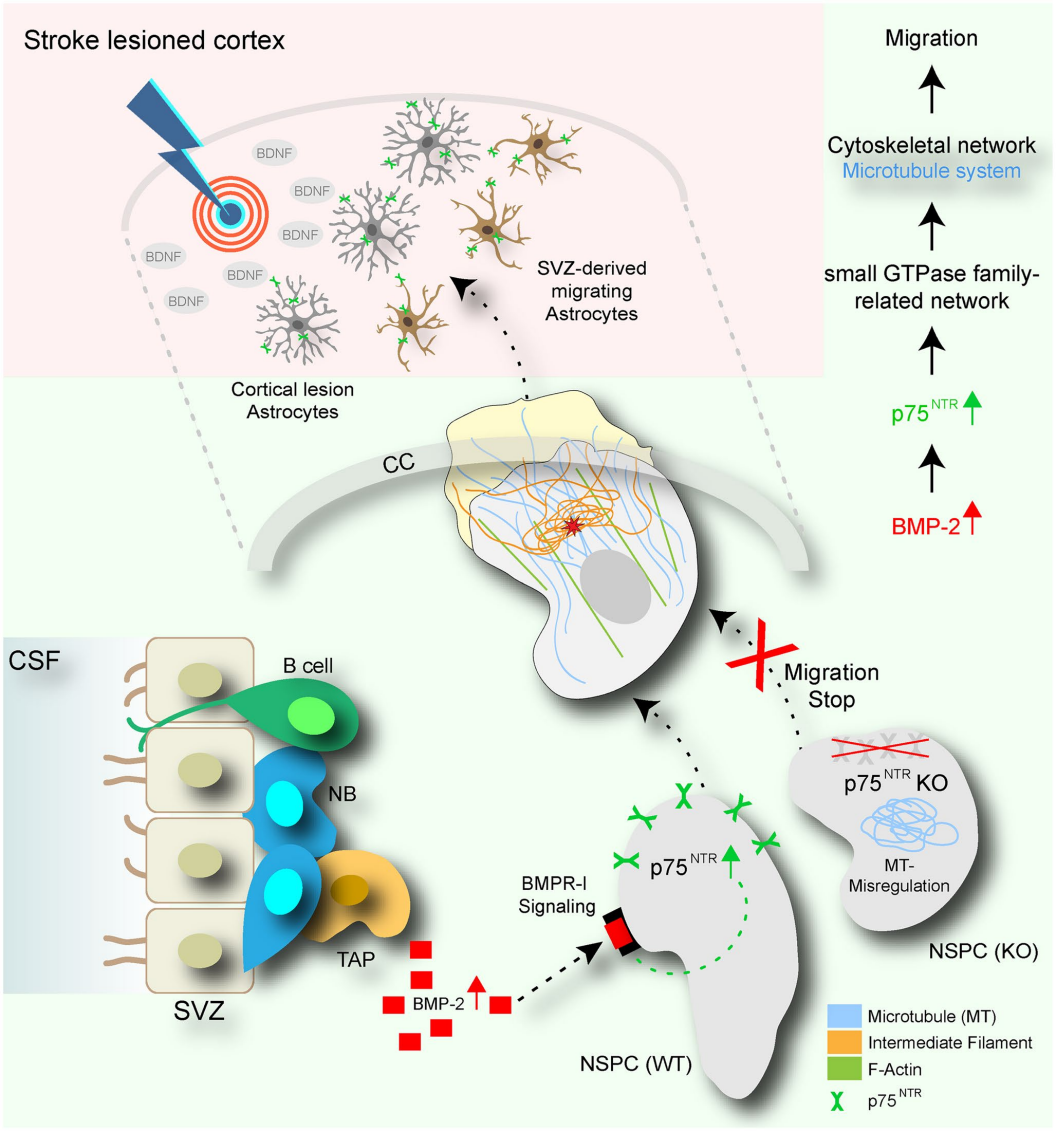
p75^NTR^ regulates SVZ NSPC migration. Cortical ischemic stroke causes a temporary BMP-2-induced p75^NTR^ upregulation by NSPCs within the SVZ stem cell niche, furnishing SVZ NSPCs migratory, potentially via regulation of the cytoskeletal network organization, enabling their redirected migration towards the cortical lesion area

In this study, we show that injury-induced BMP-2-regulated p75^NTR^ is required for the migration of SVZ NSPCs to the cortical lesion. Our results suggest the following working model in CNS disease: (i) cortical brain injury drastically changes the microenvironment of the SVZ stem cells and rapidly increases the BMP-2 level (Fig. 7). (ii) BMP receptor signaling increases the p75^NTR^ abundance in NSPCs. (iii) Increased p75^NTR^ regulates the cytoskeletal organization necessary for NSPC migration towards a BDNF gradient (Fig. 7). Collectively, BMP-induced p75^NTR^ level might be critical in regulating the migration and contribution of SVZ-derived NSPCs to contribute to brain injury sites.

Although the injury- and disease-induced p75^NTR^ expression in different tissues and cell types is well recognized and the various p75^NTR^ functions in central and peripheral tissues are well described, the regulation of p75^NTR^ expression and abundance is only poorly understood. So far, specificity protein 1 (Sp1) transcription factor has been shown to induce *p75*^*NTR*^ expression after osmotic swelling (Peterson and Bogenmann 2003; Ramos et al. 2007), early growth response factors 1 and 3 have been identified as direct modulators of p75^NTR^ (Gao et al. 2007) and *p75*^*NTR*^ is under circadian control regulated by the helix–loop–helix transcription factors CLOCK and BMAL1 (Baeza-Raja et al. 2013). In this study, we show that BMP-2 regulates p75^NTR^ mRNA and protein expression in NSPCs. BMP signaling plays a critical role in maintaining adult neural stem cell niches by promoting neurogenesis at basal level and favoring astrogliogenesis at elevated level (Bohrer et al. 2015; Bond et al. 2012; Lim et al. 2000; Pous et al. 2020). BMP in the SVZ stem cell niche is rapidly upregulated after traumatic injury (Bohrer et al. 2015) and BMP-2 is rapidly and strongly increased after photothrombotic ischemia (this study). Furthermore, vascular leakage and blood-derived fibrinogen activates BMP signaling in NSPCs (Pous et al. 2020), suggesting that changes of the SVZ stem cell niche environment in CNS injuries and diseases induce p75^NTR^ expression level via BMP signaling in NSPCs. Future studies will show whether injury-induced BMP-signaling in other cells than NSPCs will also regulate p75^NTR^ abundance.

In the brain, expression of p75^NTR^ is upregulated in astrocytes (Chen et al. 2020; Schachtrup et al. 2015) and in SVZ-derived newborn astrocytes (this study) following injury. p75^NTR^ regulates nucleocytoplasmic shuttling of the transcriptional mediator of TGF-β signaling required for glial scar formation (Schachtrup et al. 2015) and the transcriptional mediator of BMP signaling required for NSPC differentiation into astrocytes (our study). Future studies will reveal the underlying mechanism how p75^NTR^ regulates cell-specific Smad shuttling to determine differential growth factor signal transduction pathways between astrocytes and NSPCs.

Cell migration involves dynamic and spatially regulated changes to the cytoskeleton and cell adhesion, which is due largely to the regulation of Rho GTPases (Ridley 2001). A signaling relationship between p75^NTR^ and Rho is well documented in neurons, where p75^NTR^-mediated Rho signaling is involved in the regulation of neurite outgrowth (Yamashita and Tohyama 2003). Our data suggest that p75^NTR^ controls Rho GTPase activating and inhibiting proteins (Rho-GAPs and Rho-GEFs) in adult SVZ NSPCs. Rho family proteins are active in response to extracellular signals including soluble cytokines, growth factors and neurotrophins. It is therefore possible that full-length p75^NTR^ in SVZ NSPC functions as a sensor for increased cortical lesion BDNF gradients to modulate Rho family activity facilitating the SVZ NSPC cytoskeletal rearrangement necessary for migration. Yet, the underlying mechanisms how p75^NTR^ regulates Rho-GAPs and Rho-GEFs and the cytoskeletal rearrangements in NSPCs is unknown and will be an attractive future avenue.

p75^NTR^ functions at the molecular nexus of cell death, survival, and differentiation (Malik et al. 2021) and genetic loss of p75^NTR^ reduces the infarct volume in stroke (Irmady et al. 2014). Expression of p75^NTR^ is upregulated in astrocytes (Schachtrup et al. 2015) and in adult SVZ NSPCs (our study) after CNS injury. In astrocytes, increased p75^NTR^ expression level promote the reentry into the cell cycle by upregulating CDK2 expression (Chen et al. 2020) and regulate their hypertrophic reactivity (Chen et al. 2020; Schachtrup et al. 2015). Increased p75^NTR^ expression level in adult SVZ NSPCs promotes their migration towards the lesion area and their contribution to the forming scar. Future studies will identify the functional role of the newborn p75^NTR^-positive SVZ-derived astrocytes in the lesion area and whether these newborn astrocytes contribute to the regeneration process and functional recovery after stroke. Furthermore, modifying p75^NTR^ signaling in NSPCs suitable for cell transplantation might be an interesting strategy to control the cells migration and fate. Overall, the identification of BMP-2-induced p75^NTR^ abundance as a molecular link between injury-induced stem cell niche environmental changes, p75^NTR^ functions, and neural stem cell migration could provide therapeutic targets for harnessing endogenous or transplanted NSPCs for improving CNS repair in various neurodegenerative diseases.

## Supplementary Information

Below is the link to the electronic supplementary material.

Supplementary file1 (TIF 5512 kb)

Supplementary file2 (TIF 5184 kb)

Supplementary file3 (TIF 7064 kb)

Supplementary file4 (TIF 1259 kb)

Supplementary file5 (TIF 4675 kb)

Supplementary file6 (PDF 652 kb)

## Data Availability

RNA-Seq data reported in this paper are available with the GEO accession number GSE152396. Source data are provided as a source data file. All other data are available from the corresponding author upon reasonable request.
