# Supplementary figures and images for: P75 neurotrophin receptor controls subventricular zone neural stem cell migration after stroke
Source: Cell Tissue Res. 2021 Oct 26;387(3):415–31. doi: 10.1007/s00441-021-03539-z (PMC8975773; doi:10.1007/s00441-021-03539-z)

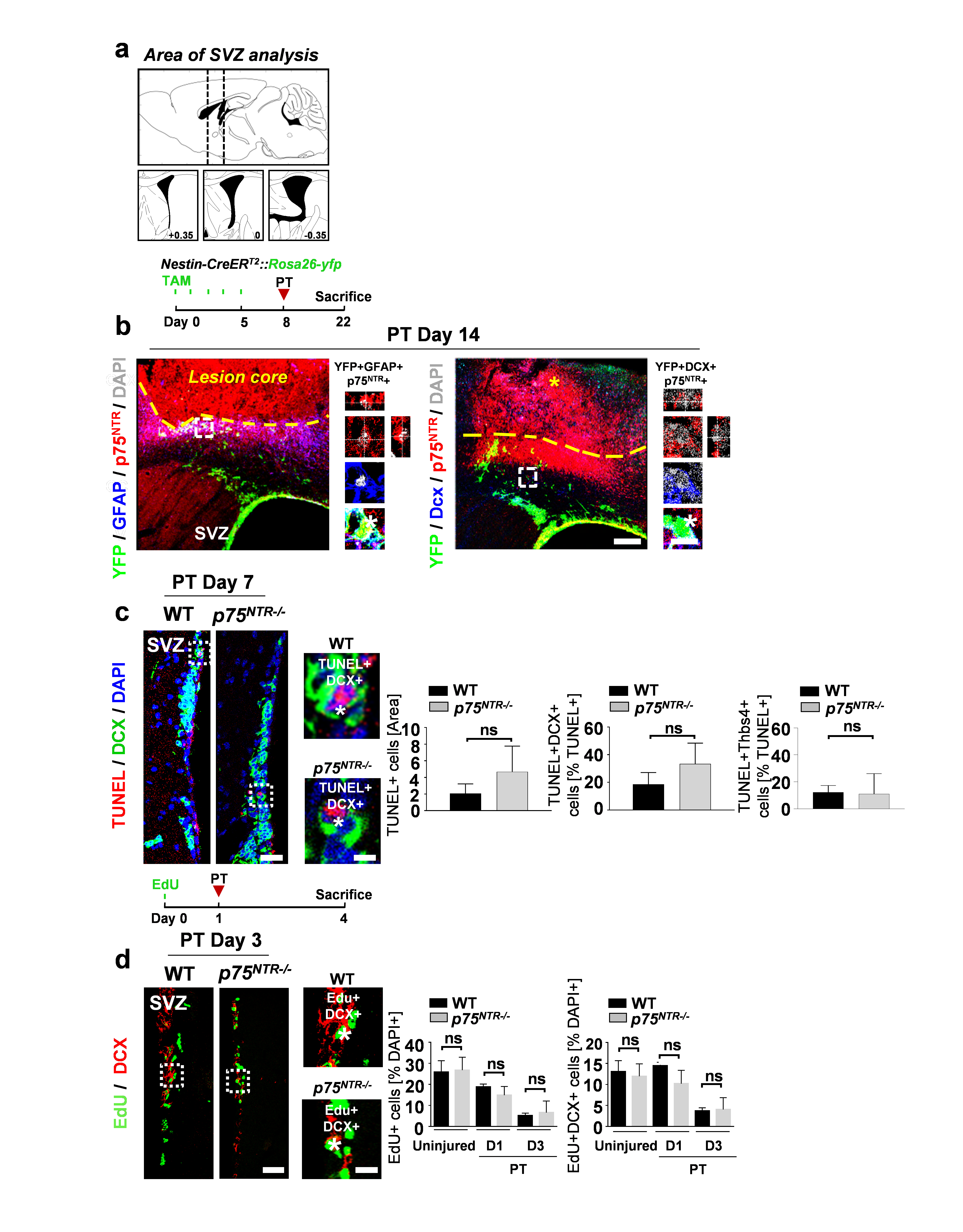

Supplement: Supplementary file 1 — Supplementary file1 (TIF 5512 kb) [file 441_2021_3539_MOESM1_ESM.tif]

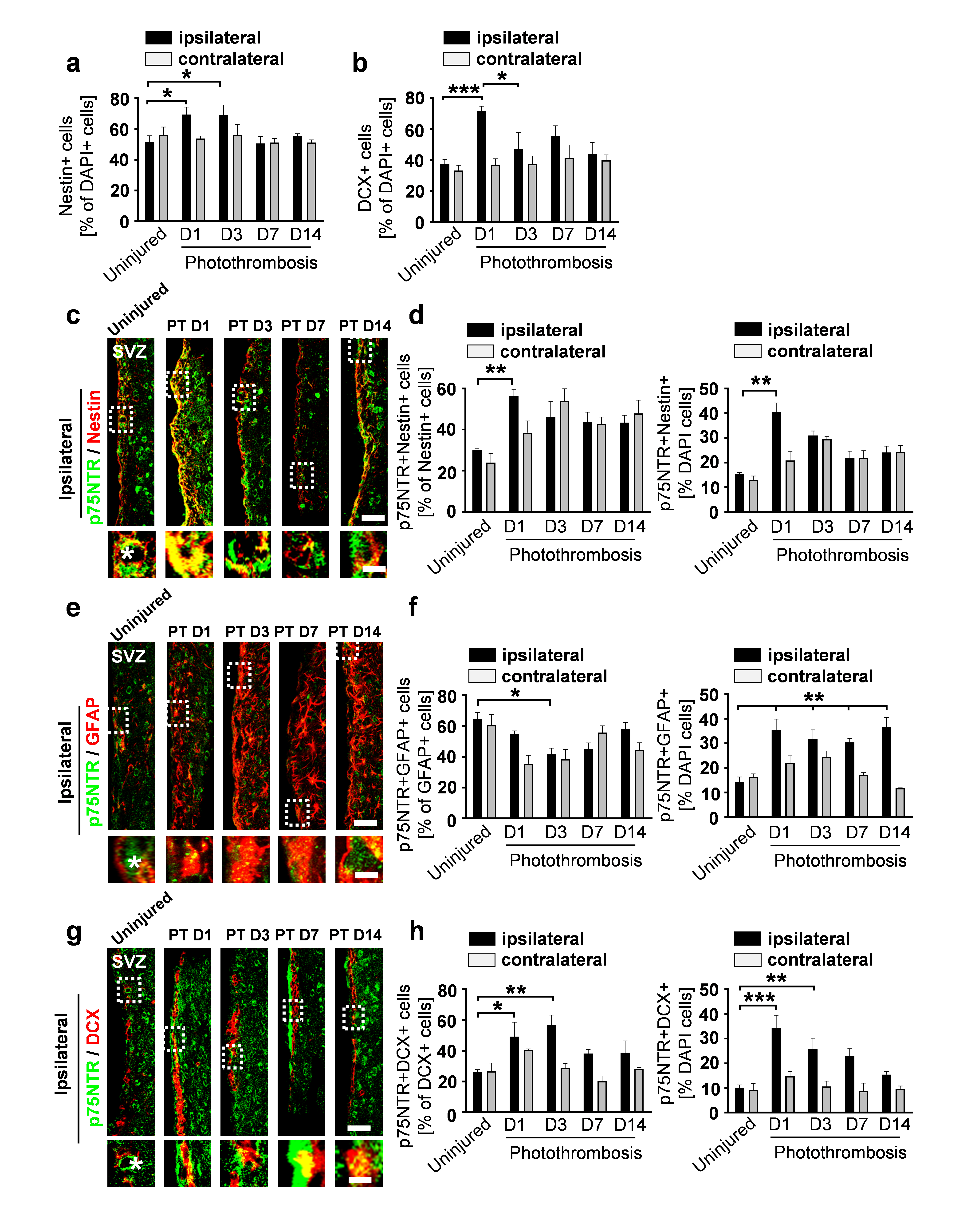

Supplement: Supplementary file 2 — Supplementary file2 (TIF 5184 kb) [file 441_2021_3539_MOESM2_ESM.tif]

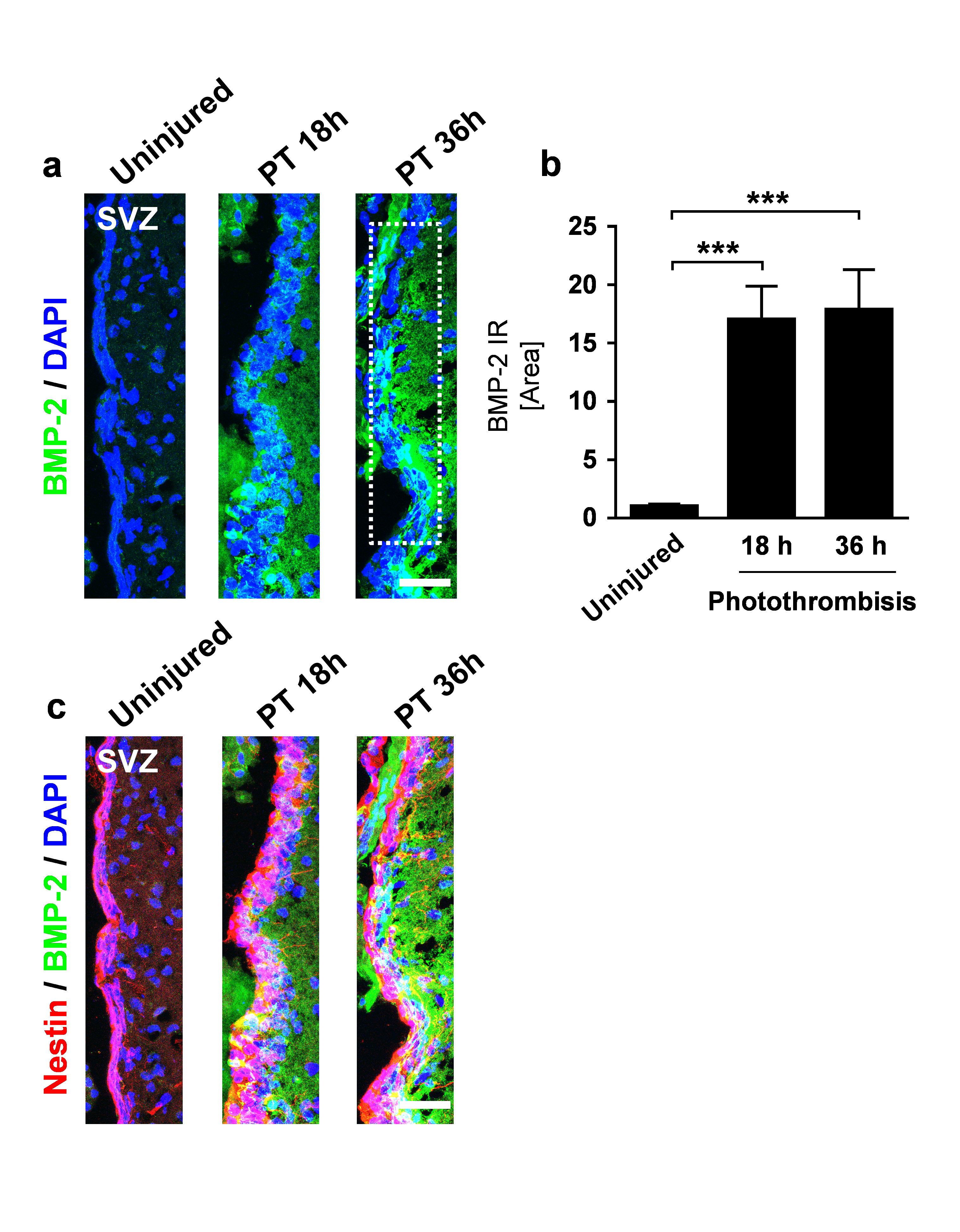

Supplement: Supplementary file 3 — Supplementary file3 (TIF 7064 kb) [file 441_2021_3539_MOESM3_ESM.tif]

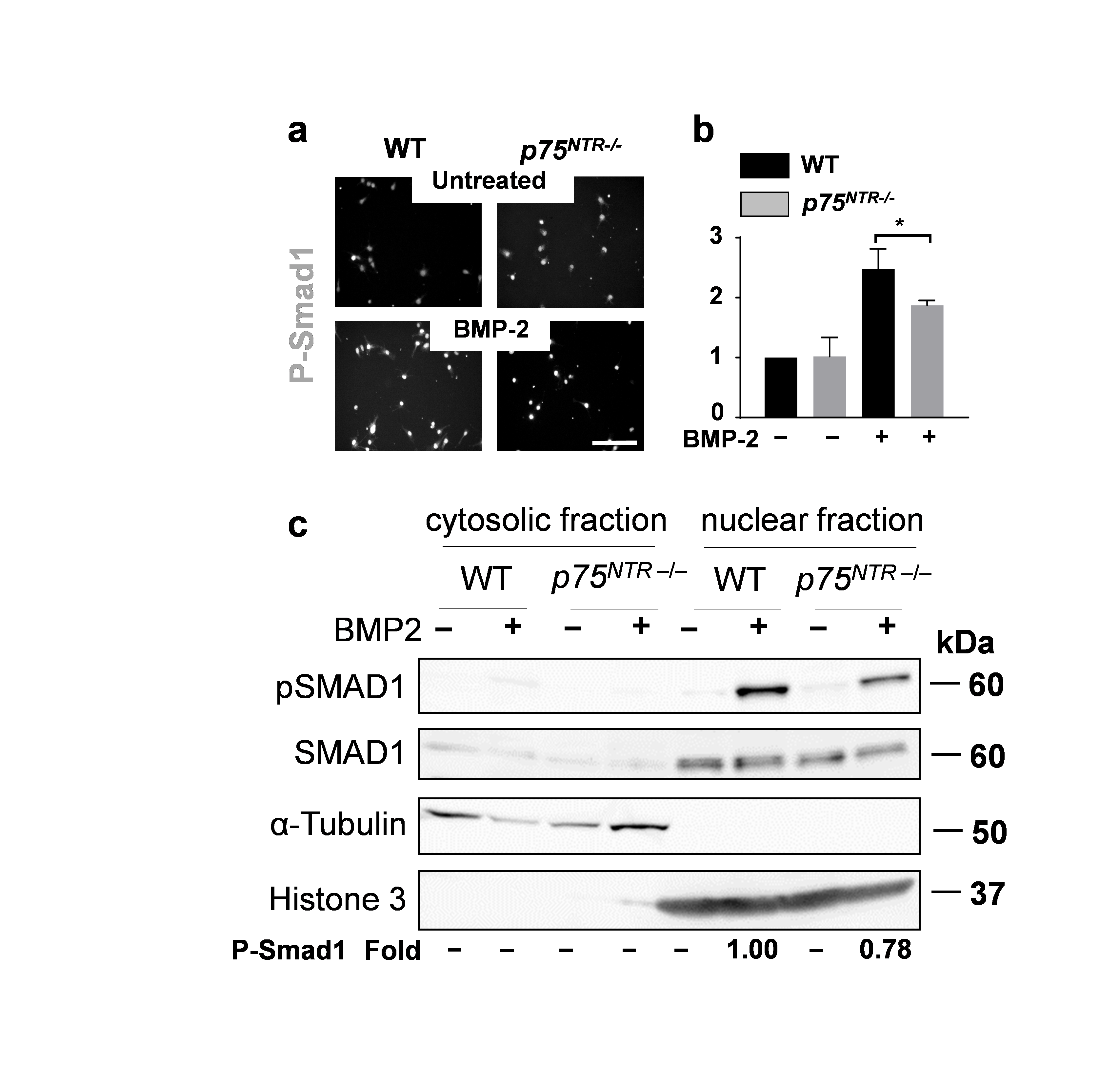

Supplement: Supplementary file 4 — Supplementary file4 (TIF 1259 kb) [file 441_2021_3539_MOESM4_ESM.tif]

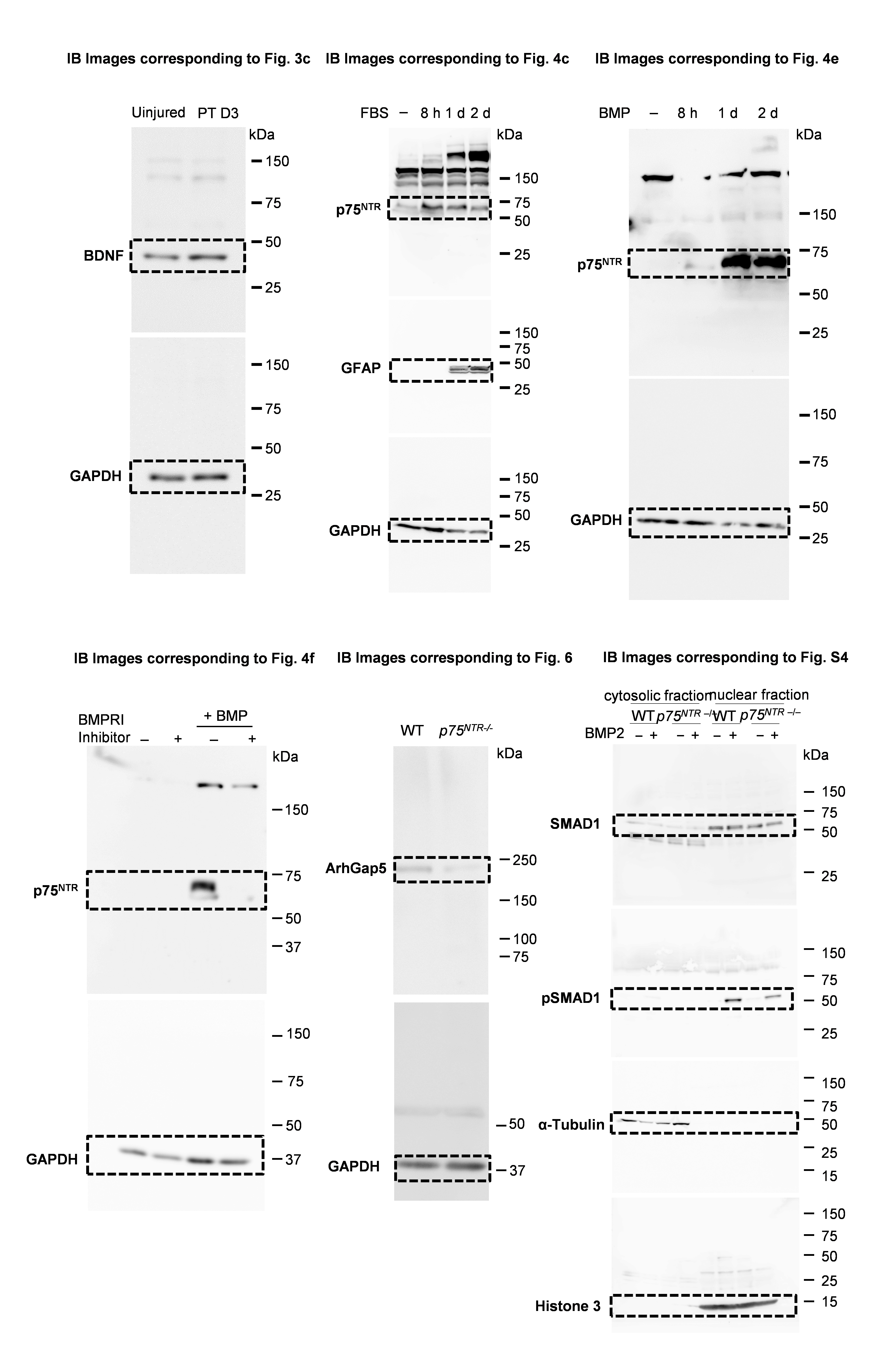

Supplement: Supplementary file 5 — Supplementary file5 (TIF 4675 kb) [file 441_2021_3539_MOESM5_ESM.tif]
