# Supplementary material for: P75 neurotrophin receptor controls subventricular zone neural stem cell migration after stroke
Source: Cell Tissue Res. 2021 Oct 26;387(3):415–31. doi: 10.1007/s00441-021-03539-z (PMC8975773; doi:10.1007/s00441-021-03539-z)
Supplement: Supplementary file 6 — Supplementary file6 (PDF 652 kb) [file 441_2021_3539_MOESM6_ESM.pdf]

Deshpande et al., Table S1

p75$^{NTR-/-}$ untreated vs. WT untreated

Rank	Gene Symbol	ENSEMBLE ID	p-value	p-value adjusted)	Fold-change
1	Dst	ENSMUST00000097785	1,14E-13	3,81E-09	-22,16976675
2	Morf4l2	ENSMUST00000152150	2,52E-05	0,007049552	-22,02155982
3	Tbc1d14	ENSMUST00000146430	3,70E-10	1,61E-06	-21,004708
4	Fam171a1	ENSMUST00000062934	5,19E-05	0,019637299	-20,92494815
5	Pcnt	ENSMUST00000001179	7,81E-07	0,001769697	-20,50042582
6	Pard3	ENSMUST00000160272	4,08E-13	3,45E-09	-20,09521478
7	Pkm	ENSMUST00000217353	0,001407604	0,120650312	-17,42798352
8	Larp4	ENSMUST00000057632	0,007738605	0,249838596	-8,784398602
9	Ctsa	ENSMUST00000103093	0,038191489	0,718738538	-8,69335327
10	Gal3st4	ENSMUST00000100530	0,004672661	0,25373636	-8,17692281
11	Aplp2	ENSMUST00000213254	0,00598963	0,400409615	-8,087600171
12	Arhgap12	ENSMUST00000062584	0,003334827	0,184770464	-7,989565517
13	Txn2	ENSMUST00000173631	0,001152498	0,136397301	-7,830703917
14	Nek4	ENSMUST00000050171	0,000766437	0,09065309	-7,8060454
15	Zfp788	ENSMUST00000100275	0,015533175	0,342347	-7,701494745
16	Vcan	ENSMUST00000159910	0,000979523	0,0719204	-7,681346402
17	Arfip2	ENSMUST00000210911	1,57E-09	4,82E-06	-7,674078684
18	Mau2	ENSMUST00000212451	0,042247768	0,622551323	-7,627671352
19	Pde4b	ENSMUST00000106904	0,024334633	0,606686975	-7,548761488
20	Tbc1d14	ENSMUST00000136189	0,032393857	0,543735832	-7,475838938
21	Trp53bp1	ENSMUST00000110647	0,010357504	0,371779033	-7,438424625
22	Plec	ENSMUST00000089610	1,62E-07	0,000698131	-7,408042508
23	Atxn7l2	ENSMUST00000119650	0,017070077	0,348540714	-7,332931049
24	Nrxn1	ENSMUST00000159778	0,002536382	0,310249617	-7,195348765
25	Cyp26b1	ENSMUST00000204146	6,36E-06	0,004478485	-7,18017217
26	Arhgef2	ENSMUST00000175911	0,013030186	0,440797142	-7,162914631
27	Reln	ENSMUST00000161356	0,000404579	0,04541447	-7,089443474
28	Cask	ENSMUST00000156096	0,008857644	0,34541437	-7,068438525
29	Med12l	ENSMUST00000040325	0,002637888	0,1701558	-7,044187927
30	Etnppl	ENSMUST00000166187	0,001679147	0,194432488	-7,034577511
31	Haus8	ENSMUST00000110071	0,000104352	0,070169583	-7,023353953
32	Dlg1	ENSMUST00000115205	0,044495018	0,590115309	-7,005683817
33	Dtd2	ENSMUST00000085404	7,31E-08	0,000250763	-6,985621374
34	Sh3bp2	ENSMUST00000101316	1,69E-07	0,000461392	-6,950608865
35	Zfp385b	ENSMUST00000111830	0,012880022	0,58515102	-6,944003234
36	Eps15	ENSMUST00000132165	1,82E-06	0,002957909	-6,919171613
37	Luc7l	ENSMUST00000140427	0,040399877	0,732646784	-6,888156266
38	Mier3	ENSMUST00000047412	0,003997822	0,23857332	-6,674493979
39	Grip1	ENSMUST00000154238	0,030334629	0,824798649	-6,647315401
40	Tfeb	ENSMUST00000024786	0,019054959	0,467932735	-6,495226663
41	Osbpl1a	ENSMUST00000155650	0,001064958	0,108716722	-6,477464153
42	Abr	ENSMUST00000108407	0,011976591	0,489715766	-6,448903964
43	Nfix	ENSMUST00000076715	0,030083255	0,440674795	-6,393776967

44	Cast	ENSMUST00000222588	0,031777165	0,520845829	-6,30644775
45	Map4k5	ENSMUST00000049239	0,01436251	1	-6,243316106
46	Flot1	ENSMUST00000173147	0,008745171	0,261017355	-6,174998368
47	Smim19	ENSMUST00000033935	0,02867431	0,528224851	-6,143977078
48	Phka1	ENSMUST00000120270	0,01924055	0,373151101	-6,125821372
49	Enah	ENSMUST00000193074	0,001675838	0,133606949	-6,063128732
50	Smarca2	ENSMUST00000099537	0,009329817	0,351296444	-6,007784707
51	Sri	ENSMUST00000088786	0,032956386	0,467401494	-5,990951067
52	Chd3	ENSMUST00000092971	0,028032671	0,497412022	-5,950744288
53	Psmc3	ENSMUST00000146506	0,002296814	0,194356057	-5,918786342
54	Rin3	ENSMUST00000056950	0,042859016	0,60780658	-5,912640228
55	Rnpepl1	ENSMUST00000179127	0,041096764	0,677391332	-5,781324026
56	Gpcpd1	ENSMUST00000145694	0,005392217	0,267968581	-5,778731849
57	Adgrl3	ENSMUST00000120673	0,025115784	0,520432344	-5,658223536
58	Tsc22d1	ENSMUST00000134109	9,30E-05	0,028696567	-5,614629806
59	Abhd3	ENSMUST00000117828	0,032670714	0,411026079	-5,566954654
60	Cacna2d1	ENSMUST00000039370	0,004168248	0,198195075	-5,556751187
61	Pax6	ENSMUST00000111086	0,032386555	0,445781447	-5,555243358
62	Col11a2	ENSMUST00000114255	0,01794039	0,498376124	-5,518439541
63	Zfp398	ENSMUST00000114598	0,022558826	0,500406149	-5,491390737
64	Rin1	ENSMUST00000025818	0,027200431	0,63039136	-5,475750004
65	Madd	ENSMUST00000066420	0,003044578	0,347984229	-5,397771645
66	Pparg	ENSMUST00000000450	0,000470873	0,120950733	-5,287321535
67	Slc24a2	ENSMUST00000107157	0,004165107	0,250657692	-5,283859783
68	Syt17	ENSMUST00000081574	0,033514327	0,56063874	-5,10335233
69	Rasgef1b	ENSMUST00000031276	0,018681093	0,458341011	-5,084001202
70	Drp2	ENSMUST00000113224	0,001391149	0,09065309	-5,016058383
71	Ikbke	ENSMUST00000062108	0,036262641	0,578701349	-4,972286094
72	S100a1	ENSMUST00000107340	0,008002089	0,497278234	-4,971698183
73	Prrc2c	ENSMUST00000028016	0,039946662	0,763082669	-4,922153015
74	Zfr	ENSMUST00000156752	0,002136077	0,286603295	-4,838821309
75	Dnmt3b	ENSMUST00000109773	0,01157719	0,42101781	-4,746980685
76	Phtf1	ENSMUST00000150849	0,004707435	0,398243521	-4,702568342
77	Hmgcs2	ENSMUST00000120541	0,000270517	0,057951795	-4,578700718
78	Mamdc4	ENSMUST00000095117	0,04875624	0,521845361	-4,483098249
79	Cdkl3	ENSMUST00000128853	0,016962947	0,595218747	-4,47806181
80	Kif13a	ENSMUST00000223881	3,44E-06	0,002053273	-4,473482639
81	Pcdha2	ENSMUST00000195590	0,001226301	0,149234338	-4,44319599
82	Pcbp2	ENSMUST00000229958	0,026309192	1	-4,37895249
83	Tti1	ENSMUST00000124338	0,039876393	0,597551652	-3,9714309
84	Tm6sf2	ENSMUST00000110160	0,030729154	0,812673787	-3,964209312
85	Cfap46	ENSMUST00000140820	0,017545698	0,536435966	-3,924259063
86	Synpo	ENSMUST00000130360	0,024254039	0,401865256	-3,85782783
87	Tm2d3	ENSMUST00000129166	0,022126602	0,585963346	-3,842966861
88	Trip11	ENSMUST00000177183	0,029512699	0,680997962	-3,717443771
89	Vwa7	ENSMUST00000007245	0,035349879	0,571860821	-3,697420625
90	Rabgef1	ENSMUST00000148264	0,013121999	0,310759673	-3,642737495
91	1700088E04Rik	ENSMUST00000190509	0,007497948	0,39081327	-3,616338327

92	Rnase1	ENSMUST00000080126	0,003894806	0,590971836	-3,353968126
93	Dst	ENSMUST00000182697	0,002898926	0,236539021	-3,296796567
94	Ablim2	ENSMUST00000129347	0,013428366	0,318342441	-3,284613817
95	Chst8	ENSMUST00000078686	0,000103105	0,030260833	-3,283359078
96	Nmnat3	ENSMUST00000112937	0,000630658	0,111485798	-3,273051286
97	Armh1	ENSMUST00000165128	0,010085829	0,350028573	-3,267524964
98	Nfib	ENSMUST00000107245	0,00429449	0,230392567	-3,21342114
99	Pitpnm2	ENSMUST00000162812	0,016405727	0,260616824	-3,204725432
100	Ift122	ENSMUST00000112923	0,008222481	0,303760849	-3,159672015

Deshpande et al., Table S2

p75NTR-/- untreated vs. WT untreated

Rank	Gene Symbol	ENSEMBLE ID	p-value	p-value (adjusted)	Fold-change
1	Tbc1d10a	ENSMUST00000041042	3,69E-06	0,002053273	21,42127082
2	Spata5	ENSMUST00000029277	3,68E-08	4,11E-05	19,57917206
3	Cyth2	ENSMUST00000056820	2,81E-08	6,12E-05	19,23763942
4	Zmynd11	ENSMUST00000154994	1,03E-08	4,11E-05	19,19151723
5	Map4	ENSMUST00000165876	8,64E-10	4,45E-06	19,18917837
6	Cds2	ENSMUST00000089461	3,21E-06	0,004192208	18,61139954
7	Rxra	ENSMUST00000100251	3,38E-05	0,022694034	18,52416227
8	Plec	ENSMUST00000169438	0,000146365	0,037267317	18,50340683
9	Tmem161a	ENSMUST00000182980	2,80E-10	1,97E-06	18,32658232
10	Cuta	ENSMUST00000114935	0,001148673	0,111055227	18,27596688
11	Efemp2	ENSMUST00000165485	2,91E-07	0,000271386	18,10890072
12	Tbc1d25	ENSMUST00000039892	0,000701853	0,116919937	18,06532646
13	Micall2	ENSMUST00000170773	4,44E-05	0,025878937	17,93713146
14	Cacna1c	ENSMUST00000189389	1,76E-09	5,54E-06	17,9291561
15	Mapk8ip3	ENSMUST00000117509	1,67E-08	4,11E-05	17,80152071
16	Ebf4	ENSMUST00000110288	0,000119979	0,046731441	17,11729539
17	Igsf8	ENSMUST00000139528	2,79E-05	0,019637299	16,87315954
18	Hp1bp3	ENSMUST00000105827	0,000304825	0,076990729	15,57068105
19	Arap1	ENSMUST00000084895	0,00214342	0,197035953	15,14864928
20	Nr2c2	ENSMUST00000113460	0,007901331	0,333521756	14,9268956
21	Sh3glb2	ENSMUST00000163668	0,008656825	0,260616824	14,75385388
22	Cul3	ENSMUST00000164108	0,000269413	0,056145509	14,3226665
23	Rit1	ENSMUST00000172252	0,007593632	0,310249617	13,93497735
24	Gm15446	ENSMUST00000112544	1,69E-11	2,80E-07	9,849991027
25	Adgrl2	ENSMUST00000196526	0,002309416	0,152814838	9,586120524
26	Ssbp4	ENSMUST00000211608	4,85E-10	1,18E-06	9,229721588
27	Golga2	ENSMUST00000081670	1,12E-06	0,000754227	8,745755793
28	Epb41l2	ENSMUST00000219900	0,025891481	0,521444658	8,727554274
29	Eif2ak4	ENSMUST00000110872	0,031961019	0,443110136	8,621096782
30	Rps9	ENSMUST00000108624	0,00404897	0,194432488	8,606224364
31	Crb1	ENSMUST00000198445	0,001968749	0,190024203	8,41586995
32	Phka2	ENSMUST00000112380	0,001017225	0,109386639	8,349113318
33	Myo18a	ENSMUST00000164334	0,004785309	0,342372795	8,348515401
34	Fgfr3	ENSMUST00000202791	2,97E-05	0,026957645	8,342485921
35	Guk1	ENSMUST00000170895	5,69E-06	0,029269848	8,313679483
36	Cdk10	ENSMUST00000213005	6,02E-06	0,003930647	8,29690451
37	Ddhd1	ENSMUST00000111828	0,000684985	0,076990729	8,259175352
38	Crtc2	ENSMUST00000184882	7,36E-08	0,000138278	8,24383805
39	Psmd4	ENSMUST00000140348	0,026615708	0,528224851	8,237898336
40	Fgfr1	ENSMUST00000167764	0,005244486	0,218848474	8,182290348
41	Ciz1	ENSMUST00000113331	8,84E-05	0,027154132	8,117430281

42	Mapk8ip3	ENSMUST00000178969	0,002353438	0,115468311	8,088381683
43	Syde2	ENSMUST00000039517	0,012521207	0,394222165	8,061865075
44	Dhx57	ENSMUST00000038166	0,000993239	0,072187586	8,004740914
45	Mta3	ENSMUST00000112352	9,10E-06	0,003945108	7,932019708
46	Btbd6	ENSMUST00000002880	0,004343532	0,154870238	7,853525862
47	Npepps	ENSMUST00000165216	0,014227164	0,328985855	7,821759918
48	Smarcd2	ENSMUST00000106843	7,31E-07	0,007108796	7,759625432
49	Ckap5	ENSMUST00000111337	0,009666199	0,268451793	7,75903256
50	Hdac5	ENSMUST00000107151	1,71E-06	0,001175009	7,751801791
51	Mkrn1	ENSMUST00000031985	0,026049435	0,513432116	7,690699535
52	Igfbp4	ENSMUST00000177092	0,002228249	0,232943657	7,690310656
53	Mark2	ENSMUST00000025921	0,029370196	0,533478804	7,665566896
54	Rbm6	ENSMUST00000035201	0,018520817	0,377997536	7,662750643
55	Zwilch	ENSMUST00000176794	0,014284935	0,411920857	7,593172681
56	Men1	ENSMUST00000078137	0,001722173	0,183082726	7,591315894
57	Vps29	ENSMUST00000117868	0,000205542	0,064426169	7,560354285
58	Tardbp	ENSMUST00000185673	0,002970177	0,179739014	7,552914548
59	Ddit4l	ENSMUST00000165845	2,77E-05	0,014524687	7,506622437
60	Adcy6	ENSMUST00000096224	0,005404795	0,220216103	7,460938156
61	Slc15a2	ENSMUST00000168279	1,36E-06	0,002430808	7,448630179
62	Kdm2b	ENSMUST00000156474	0,007285399	0,303760849	7,419678994
63	Tipin	ENSMUST00000215031	0,001840533	0,201213695	7,414967135
64	Ppip5k1	ENSMUST00000052029	4,10E-05	0,010390408	7,413999561
65	Itprid2	ENSMUST00000111784	0,007011374	0,240912263	7,395725344
66	Fip1l1	ENSMUST00000113534	0,001727005	0,176021598	7,366820685
67	Myh14	ENSMUST00000208200	0,013482192	0,314840452	7,3617061
68	Tlk2	ENSMUST00000092537	0,015198963	0,41596464	7,350498204
69	Prx	ENSMUST00000125990	0,000631277	0,109386639	7,293144073
70	Malt1	ENSMUST00000224056	0,001452811	0,169655759	7,264594705
71	Csnk1g1	ENSMUST00000206048	1,51E-05	0,00471533	7,264485507
72	Mark2	ENSMUST00000171721	0,032535802	0,564130124	7,238243661
73	Abcc10	ENSMUST00000047970	0,000588608	0,080470854	7,234069222
74	Nedd4	ENSMUST00000184450	0,030665746	0,550256826	7,22938606
75	Enox2	ENSMUST00000114911	0,000452991	0,0719204	7,207464685
76	Txnrd2	ENSMUST00000205679	0,007609943	0,249311951	7,192112258
77	Myt1	ENSMUST00000108757	0,023584737	0,508886388	7,1884435
78	Wnt7b	ENSMUST00000229495	7,18E-05	0,015265631	7,169795756
79	Snrpb2	ENSMUST00000126763	0,005789265	0,224785171	7,167041036
80	Taf12	ENSMUST00000030731	0,000220725	0,066866748	7,163143958
81	Cdyl	ENSMUST00000075220	7,66E-06	0,007614581	7,145697943
82	Arfrp1	ENSMUST00000185118	0,002613858	0,211442185	7,136950782
83	Ubap2l	ENSMUST00000197903	0,028976771	0,528355944	7,048934844
84	Mink1	ENSMUST00000079244	0,000683304	0,090432846	7,042723141
85	Ing3	ENSMUST00000115389	0,000100936	0,030068794	7,02124092
86	Rabep1	ENSMUST00000178245	0,028542374	0,634172726	7,013175332
87	Cacna1c	ENSMUST00000219833	0,00216307	0,111055227	6,989539433
88	Cox19	ENSMUST00000198966	3,81E-06	0,003408967	6,987727572
89	Preb	ENSMUST00000202567	0,00079509	0,124362875	6,985062289

90	Osbpl7	ENSMUST00000168565	0,027650299	0,633028921	6,98277981
91	Txndc16	ENSMUST00000147853	0,003165377	0,224568655	6,981186437
92	Usp4	ENSMUST00000194224	0,026756827	0,519388879	6,970439856
93	Emc1	ENSMUST00000082262	0,000983912	0,108101216	6,968491347
94	Fam219a	ENSMUST00000108049	0,037325271	0,584141338	6,964733741
95	Ppfia1	ENSMUST00000182226	9,43E-05	0,028447199	6,961894634
96	Mcf2	ENSMUST00000101531	0,023181297	0,543735832	6,950565891
97	Dmxl2	ENSMUST00000123709	0,023236369	0,396962008	6,948739029
98	Hnrnpab	ENSMUST00000101249	0,000402539	0,133606949	6,928635958
99	Plekhg2	ENSMUST00000121085	5,29E-05	0,019637299	6,925284511
100	Klhl18	ENSMUST00000198400	0,002033545	0,187146014	6,917654125
